# Methylome and transcriptome maps of human visceral and subcutaneous adipocytes reveal key epigenetic differences at developmental genes

**DOI:** 10.1038/s41598-019-45777-w

**Published:** 2019-07-02

**Authors:** Stephen T. Bradford, Shalima S. Nair, Aaron L. Statham, Susan J. van Dijk, Timothy J. Peters, Firoz Anwar, Hugh J. French, Julius Z. H. von Martels, Brodie Sutcliffe, Madhavi P. Maddugoda, Michelle Peranec, Hilal Varinli, Rosanna Arnoldy, Michael Buckley, Jason P. Ross, Elena Zotenko, Jenny Z. Song, Clare Stirzaker, Denis C. Bauer, Wenjia Qu, Michael M. Swarbrick, Helen L. Lutgers, Reginald V. Lord, Katherine Samaras, Peter L. Molloy, Susan J. Clark

**Affiliations:** 1 Epigenetics Research Laboratory, Genomics and Epigenetics Division, Garvan Institute of Medical Research, Sydney, 2010 New South Wales Australia; 2 CSIRO Health & Biosecurity, North Ryde, 2113 New South Wales Australia; 3 St Vincent’s Clinical School, UNSW, Sydney, 2010 New South Wales Australia; 4 CSIRO Data61, North Ryde, 2113 New South Wales Australia; 5 Department of Biological Sciences, Macquarie University, North Ryde, 2019 New South Wales Australia; 6 Centre for Diabetes, Obesity and Endocrinology, The Westmead Institute, The University of Sydney, Westmead, 2145 New South Wales Australia; 7 Department of Endocrinology, University of Groningen, University Medical Center Groningen, Groningen, the Netherlands; 8 St. Vincent’s Centre for Applied Medical Research, Sydney, 2010 New South Wales Australia; 9 Department of Endocrinology, St Vincent’s Hospital, Sydney, 2010 New South Wales Australia; 10 Diabetes and Metabolism Division, Garvan Institute of Medical Research, Sydney, 2010 New South Wales Australia

**Keywords:** DNA methylation, Cell biology, Endocrine system and metabolic diseases

## Abstract

Adipocytes support key metabolic and endocrine functions of adipose tissue. Lipid is stored in two major classes of depots, namely visceral adipose (VA) and subcutaneous adipose (SA) depots. Increased visceral adiposity is associated with adverse health outcomes, whereas the impact of SA tissue is relatively metabolically benign. The precise molecular features associated with the functional differences between the adipose depots are still not well understood. Here, we characterised transcriptomes and methylomes of isolated adipocytes from matched SA and VA tissues of individuals with normal BMI to identify epigenetic differences and their contribution to cell type and depot-specific function. We found that DNA methylomes were notably distinct between different adipocyte depots and were associated with differential gene expression within pathways fundamental to adipocyte function. Most striking differential methylation was found at transcription factor and developmental genes. Our findings highlight the importance of developmental origins in the function of different fat depots.

## Introduction

Adipocytes are the predominant cell type of adipose tissue and central to its key metabolic and endocrine functions, including storing or releasing triglycerides, and secreting adipokines that regulate metabolic, hormonal and inflammatory pathways^1,2^. In mammals lipid is primarily stored in two major classes of depot, visceral adipose depots and subcutaneous adipose depots^2,3^. While the different depots share many properties, there are important functional differences between visceral adipose tissue (VAT) and subcutaneous adipose tissue (SAT). These include differences in lipolysis, insulin sensitivity, adipokine secretion and inflammatory and immune function^4,5^. Increased visceral adiposity has been associated with many of the poor health outcomes linked to obesity^6,7^: type 2 diabetes mellitus, heart disease, and certain types of cancer^8,9^, as well as increased mortality^10^. In contrast, SAT, particularly lower-body SAT, is considered relatively metabolically benign^11,12^. However, molecular features associated with the functional differences between these similar, yet distinct, cell types are not well understood.

The epigenome is essential in enforcing, and sequentially restricting cell specific expression patterns throughout human development, and in maintaining cells in their appropriate functional states. The advent of next-generation sequencing technologies has enabled the creation of epigenome-wide data sets to provide insights into the molecular characteristics that underpin differences in normal cell function. As adipocytes from visceral and subcutaneous depots (VA and SA respectively) perform many of the same functions, we were interested to compare the epigenomes and transcriptomes of these similar yet distinct cell types. Comparative genome-wide studies have previously used microarray-based analysis of whole adipose tissues which are a mixture of different cell types^13–15^. Whole methylome analysis of subcutaneous tissue^16^ or capture bisulphite sequencing of visceral tissue^17^ have been reported separately for each tissue type but not for isolated cells. Here, we isolated adipocytes from matched SAT and VAT of individuals with a normal BMI to comprehensively characterise each of the transcriptomes and DNA methylomes to determine the epigenetic differences and their potential contribution to cell type and depot-specific function. Our results show that the cell-type specific DNA methylation profiles were associated with differential gene expression within pathways fundamental to adipocyte function and highlight the importance of developmental origins in the function of different fat depots.

## Results

### Distinct DNA methylomes and transcriptomes characterise visceral and subcutaneous adipocytes

**Figure 1:**
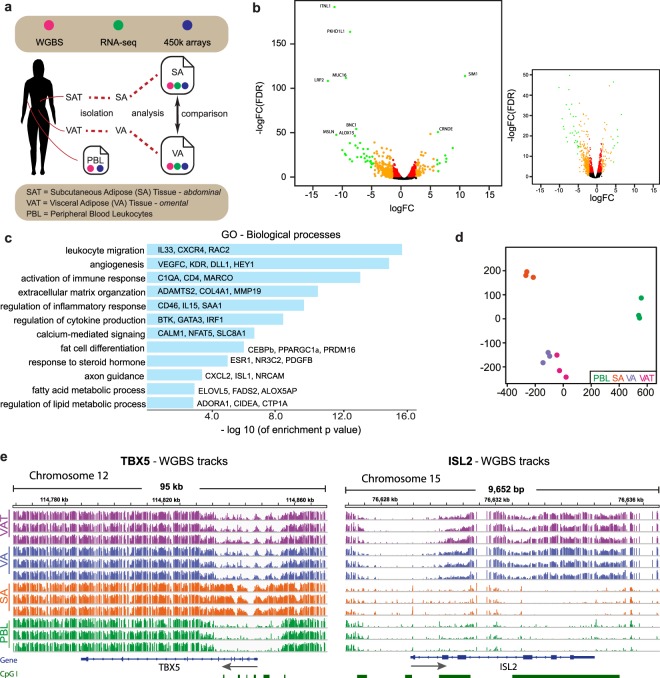
Methylomes of adipocytes cluster based on fat depot. **(a)** Diagrammatic representation of samples taken and study design **(b)** Volcano plot of differences in expression between SA and VA in whole RNA-seq. (**main**) Red points FDR < 0.001, orange points logFC >2, green points FDR < 1e-6 and logFC >5. (**right box**) Points with FDRs less than 1e-50 removed, orange dots FDR < 0.001 and logFC >2. **(c)** Gene ontology of biological processes enriched in genes differentially expressed between SA and VA. Selected gene symbols of genes DE in each term displayed. Differential expression was identified using whole RNA-seq. X-axis: Negative log10 FDR enrichment. **(d)** Multi-Dimensional Scaling (MDS) of WGBS samples from each patient based on methylation at all (17,483,788) CpGs with 5x coverage. **(e)** Browser shots of WGBS methylation levels across *TBX5* and *ISL2* loci for VAT, VA, SA and PBL samples from all patients. Arrows indicate direction of transcription. Green bars indicate CpG Islands. SAT = subcutaneous adipose tissue, SA = isolated subcutaneous adipocytes, VAT = visceral adipose tissue, VA = isolated visceral adipocytes, PBL = peripheral blood leukocytes, WGBS = whole genome bisulphite sequencing, 450 K = Illumina Infinium Human Methylation 450 BeadChip arrays, whole RNA-seq = Ribosomal RNA reduced RNA-seq. CpGI = CpG Islands, FDR = False Discovery Rate, LogFC = log of fold change.

To characterise key differences in the epigenomes of adipocytes from different depots we generated comprehensive epigenome and transcriptome maps of isolated subcutaneous and SA and VA. Whole genome bisulphite sequencing (WGBS) and stranded RNA sequencing (RNA-seq) was performed on isolated and matching SA and VA (Fig. 1a) from a core set of three females with a healthy body weight. Comparison with public methylome microarray data on whole adipose tissues from a larger cohort^18^ indicates that the three individuals are representative of normal weight subjects (Supplementary Information). Supplementary Table S1 summarises the WGBS (>25X coverage), RNA-seq data and patient characteristics, including blood biochemistry. SA and VA were also compared to whole VAT and peripheral blood leukocytes (PBL). WGBS methylation data analysis was further supported by Illumina Infinium Bead Chip Human 450 arrays (450 K arrays) on five individuals (Supplementary Information and Supplementary Table S9).

We first identified genes that were differentially expressed between SA and VA from the RNA-seq data. A total of 2,943 genes were identified, 1,582 down and 1,361 up in matched SA compared with VA (Supplementary Tables S2, S3). Greater than 91% of the differentially expressed genes were protein coding, with 1,412 down regulated and 1,281 up regulated in SA compared with VA. Figure 1b shows a volcano plot of gene expression (see also Supplementary Information). The expression profiles of isolated adipocytes are in broad agreement with differential gene expression profiles of VAT and SAT using microarray platforms^13^. Genes important in endocrine function, including *ITLN1* (Omentin) (Fig. 1b)^19^, *CCL3* (MIP-1a) and *TNF* were elevated in expression in VA, and Leptin (*LEP*) and Osteonectin (*SPARC*) higher in SA. A number of genes involved in the insulin signalling pathway also show high differential expression, including the lncRNA *CRNDE* (Fig. 1b) and *RBP4* (Retinol binding protein 4) more highly expressed in SA and *PTPN6* and *PTPRD* more highly expressed in VA (Supplementary Tables S2, S3). Pathway analysis using GOseq for the differentially expressed genes identified 1,466 enriched terms (FDR, *p* < 0.05) (Fig. 1c, Supplementary Table S4). Notably many of the highly enriched terms highlighted characteristics that are known to differ between the two adipose depots, including regulation of cytokine production, extracellular matrix, fat cell differentiation, fatty acid metabolic process, regulation of lipid metabolic process, innervation (axon guidance) and angiogenesis (Fig. 1c and Supplementary Table S4). Functions such as adipokine secretion and lipid storage are recognised intrinsic properties that distinguish visceral and subcutaneous adipocytes. However, the data further indicate that many of the gene expression differences between the isolated adipocytes also relate to whole tissue functions such as leukocyte migration, angiogenesis and extracellular matrix.

Second, analysis of WGBS data (Fig. 1d), and using the 450 K methylome data for validation (Supplementary Fig. S1), demonstrated a clear clustering by DNA methylation for each cell type. In the first dimension adipocytes and PBL separate, while SA and VA separate in the second dimension. Across the genome we found that SA and VA are hypomethylated relative to PBL, especially at CpG shores and non-island regions (Supplementary Information and Supplementary Fig. S1). Interestingly, purified VA cells and corresponding tissue (VAT) cluster closely even though VAT is a mixture of different cell types. Comparison with published methylation data on adipose tissue also show that SA and VA segregate with SAT and VAT respectively (Supplementary Information and Supplementary Fig. S1). Consistent with the tight clustering within cell types, individual methylation profiles also show high concordance between subjects (Fig. 1e). Analysis of the 450k array data demonstrates that variance was exceptionally low genome-wide within each cell type across individuals (median Standard Deviation of individual CpG sites ~1.5%, Supplementary Fig. S2). Exemplary distinct VA and SA DNA methylation profiles of *TBX5*, which is implicated in early steps of adipocyte differentiation and *ISL2*, a homologue of adipogenic factor *ISL1*, are shown in Fig. 1e (WGBS) and Supplementary Fig. S2 (450 K). Notably the promoter associated *TBX5* CpG island is hypermethylated in SA relative to VA, VAT and PBL, whereas for *ISL2* VA and VAT display high gene body methylation relative to SA and PBL.

**Figure 2:**
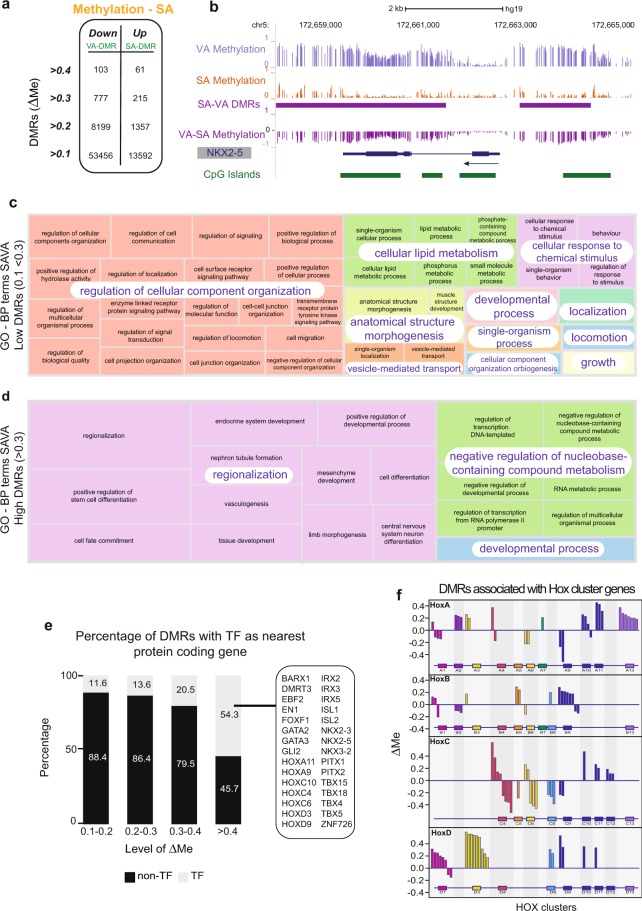
Magnitude of differential methylation between SA and VA correlates with different gene functions. **(a)** Stratification of Differentially Methylated Regions (DMRs) between SA and VA (SAVA DMRs) by average difference in methylation across the region (ΔMe), **Down** = lower methylation in SA compared to VA (VA-DMRs) and **Up** = higher methylation in SA compared to VA (SA-DMRs). **(b)** UCSC browser image of *NKX2-5* gene. Tracks include WGBS methylation tracks for VA, SA, VA-SA differential methylation, SA-VA DMR, CpG islands. **(c,d)** Overarching Gene Ontology Biological Processes terms generated using Revigo^52^ based on top 100 GO terms generated in custom GOseq (see methods) **(c)** Biological processes terms based on SAVA DMRs 0.1 to <0.3 **(d)** Biological processes terms based on SAVA DMRs >0.3. **(e)** Percentage of DMRs with transcription factors as nearest protein coding gene. X-axis = genes stratified by absolute value of ΔMe, y-axis = the percentage of DMRs that have a transcription factor as nearest protein coding gene (TF) or DMRs that do not have a transcription factor as nearest protein coding gene (non-TF). Table shows examples of transcription factors that are the closest protein coding gene to DMRs with absolute ΔMe >0.4. **(f)** Diagrammatic representation of DMRs within the HOX gene clusters. Y-axis = ΔMe, x-axis = HOX clusters.

Third, to explore how the differences in methylation profiles relate to the functional characteristics of VA and SA, we used BSmooth^20^ to identify Differentially Methylated Regions (DMRs) in WGBS data. Strikingly, we found 67,048 DMRs with a ≥10% difference in methylation averaged across the region (ΔMe of ≥0.1). Most of the DMRs (53,456) displayed hypermethylation in VA (VA-DMR), while 13,592 were hypermethylated in SA (SA-DMR) (Fig. 2a, genomic regions listed in Supplementary Tables S7 and S8). DMRs identified using 450 K arrays showed high concordance with WGBS DMRs (Supplementary Information and Supplementary Table S9). In total the WGBS DMRs encompass ~43 Mb or 1.4% of the genome. The majority of SA- and VA-DMRs were located either within gene bodies (~40%) and promoter regions (+/− 2 kb of Transcription start sites (TSS) 20%), Supplementary Fig. 3. With a more stringent DNA methylation distinction (ΔMe ≥0.3), 777 VA-DMRs and 215 SA-DMRs were identified. These “High DMRs” were found closer to the TSS of genes and had higher CpG density; there was a strong trend for increasing overlaps with CpG islands and shores with increasing ΔMe (χ^2^ 292.3 and 233.6 respectively, p < 22.e-16) with 4.5% and 27.8% of ≥0.3 DMRs overlapping CpG Islands or Shores respectively (Supplementary Fig. S3). An example of DMRs at the *NKX2-5* locus is shown in Fig. 2b. We also examined whether single nucleotide polymorphisms (SNPs) that have been associated with either fat distribution (waist-hip ratio) or obesity were enriched in regions of SA-VA DMRs. A marginally significant association of DMRs with body fat distribution SNPs was identified^21^, but none was seen with obesity SNPs^22^ (Supplementary Information).

### DNA methylation profiles highlight different developmental origins

To identify how DNA methylation is associated with the functional differences between SA and VA, we used Gene Ontology (GO) analysis to identify pathways enriched for regions that were differentially methylated between SA and VA. Using GOseq we found enrichment for terms relating to cell function, as well as development (Supplementary Fig. S4). We next examined the GO of DMRs of either Low (Low DMRs, 0.1 < ΔMe < 0.3) or High (High DMRs, ΔMe ≥ 0.3) magnitude and found a striking split in the terms that were enriched (Fig. 2c,d). Low DMRs (n = 66,056) were enriched for genes in pathways relating to cell function and enzyme activity (Fig. 2c, Supplementary Table S10). In contrast, for the 992 High DMRs a striking enrichment for DNA binding and developmental processes related to regionalisation was seen (Fig. 2d, Supplementary Table S11). A similar dichotomy was reflected in the enriched GO Molecular Function and Cellular Compartment terms (Supplementary Fig. S4).

Given the enrichment of terms relating to DNA binding in the GO analysis we compared DMR location relative to transcription factor (TF) genes^23^ and found that DMRs associated with TF genes tend to have larger ΔMe than all DMRs (Fig. 2e, Supplementary Table S12). Indeed 54.3% of highest DMRs have a transcription factor as their nearest protein-coding gene (Fig. 2e, trend χ^2^ 360.7, p < 2.2e-16). Notably, many of these TFs are essential for embryonic organization, driving cell type specification and controlling positional identity during embryonic development. For example we found that 31 of the 39 protein coding *HOX* genes^24^ were associated with DMRs (Fig. 2f – Supplementary Fig. S5). A number of the highest DMRs between SA and VA were in other gene families important in establishing cell identity and the organisation of mesodermally derived tissues, such as adipose tissue. These included the developmental transcription factors: *PITX* (1 and 2), *TBX* (4, 5, 15, 18), *IRX* (2, 3, 5) and *NKX* (2–3, 2–5, 3–2) (Supplementary Fig. S6)^25–29^. Recent 450 K data on adipose tissues has also identified enrichment of DMRs associated with developmental genes^18^. Our analysis of isolated adipocytes expands this set considerably and provides the base pair resolution necessary to characterise potential regulatory elements.

Together these findings highlight the enrichment of distinct DMRs close to developmental transcription factors and support the finding that visceral and subcutaneous depots may have different developmental origins^30^.

### Differential DNA methylation between SA and VA is associated with differential expression of development genes and genes important for adipocyte function

**Figure 3:**
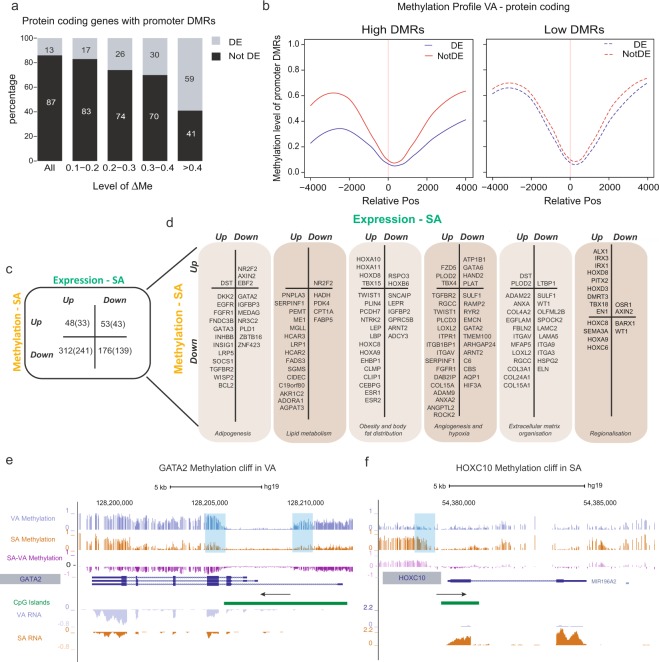
Differential DNA Methylation associated with differential gene expression. **(a)** Barplot of protein coding genes stratified according to those with promoter DMRs between 0.1 to <0.2, 0.2 to <0.3, 0.3 to <0.4, >0.4 and all protein coding genes (All). χ^2^ trend was calculated across the four ΔMe groups. The percentage of these genes are then split by whether they are differentially expressed (DE – grey) or not differentially expressed (Not DE – black). Y-axis = percentage DE or Not DE, x – axis gene stratification. **(b)** Plot of methylation level in VA across promoters containing DMRs. Loess regression was used to fit a curve representing overall methylation levels in VA, 4 kb either side of the TSS, of four groups of genes. Left panel shows plots for promoters containing DMRs with ΔMe ≥ 0.3 and right panel those with ΔMe = 0.2 to <0.3. Profiles for DE genes are shown in red and for non-DE genes in blue. X-axis = relative position to the TSS of genes, y-axis = methylation level. (**left**) DMRs – Δme ≥ 0.3**, (right)** DMRs - ΔMe 0.2 to <0.3. **(c)** Table enumerating, into four groups, differentially methylated regions within 2 kb of TSS (>0.2 ΔMe) associated with differentially expressed genes. Grouping is by differential methylation, Up or Down, with respect to SA, and differential expression, Up or Down, with respect to SA. The numbers of genes associated with DMRs are shown in brackets; in seven cases a DE gene is associated with both SA- and VA-DMRs. **(d)** Examples of genes identified, and grouped, by differential expression and methylation, as in figure (c). Genes are categorized into columns of biological processes with which they are associated. **(e) (f)** UCSC browser image of GATA2 and HOXC10. Tracks include WGBS methylation tracks for VA, SA, VA-SA differential methylation, CpG islands, and paired RNA-seq for SA and VA Blue columns highlight DNA methylation cliffs.

Integrating differential gene expression with differential methylation, we identified that ~66.1% of the differentially expressed genes had DMRs within their gene body and/or promoter. The greater the magnitude of the DMR the more likely the gene was to be differentially expressed (Supplementary Fig. S8). This relationship was particularly apparent for DMRs in the promoters of protein coding genes (Fig. 3a, trend χ^2^ 154.5, p < 2.2e-16). Interestingly, the promoter regions of differentially expressed protein coding genes with High DMRs are less methylated than the promoters of other protein coding genes (Fig. 3b and Supplementary Fig. S8). In fact, 56% of High DMRs that are within the promoters of differentially expressed protein coding genes are in regions where one cell type is lowly methylated (average methylation ≤ 0.25). In contrast only 9% of the remaining promoter DMRs are in regions where one cell type has average methylation ≤ 0.25 (χ^2^ 67.6, p = 1.37e-14). Of these High DMRs associated with protein-coding gene promoters, 64.4% overlap with CpG island shores compared with 4.7% located in islands themselves. In general, these High DMRs can visually be equated to “cliffs” rising from a methylation plain rather than “ravines” from a plateau (Fig. 3e,f, Supplementary Fig. S9). Consistent with this, High DMRs within the promoters of differentially expressed genes were preferentially located in DNA Methylation Valleys (DMV, Supplementary Fig. S8 and Supplementary Information). Thus, SAVA DMRs present in CpG shores often display a cliff profile flanking the TSS, with sharp elevation in methylation on one or both sides of an unmethylated CpG island.

To better understand how differences in DNA methylation between SA and VA may contribute to adipocyte function we focused on genes that were differentially expressed between SA and VA and that were associated with promoter DMRs (DE-DM genes). Here we chose a threshold (ΔMe ≥ 0.2) to focus on those DMRs that were more likely to have a regulatory function. In total 448 differentially expressed genes, associated with 589 DMRs, were identified and manually curated (Supplementary Tables S13 and S14, Fig. 3d). We divided our DE-DM genes into four categories based on the direction of their difference in expression and methylation between VA and SA (Fig. 3c). Many of these genes were involved in processes important for adipocyte functioning (Fig. 3d). These included genes involved in processes that display functional differences between SAT and VAT, including in lipid droplet formation and lipid mobilisation, angiogenesis and extracellular matrix remodelling. Specific examples are discussed further in Supplementary Information and Supplementary Table S14. Remarkably we found that 13 DE-DM genes were among the 43 identified by Keller *et al*. in their array-based comparison of 18 non-obese subjects^15^, despite the differences in technology and our data deriving from isolated adipocytes, rather than adipose tissue. Numerous genes with polymorphisms linked to fat distribution and waist-hip ratio were also differentially expressed and associated with promoter DMRs (Fig. 3d and Supplementary Table S14); these included the developmental genes *HOXA11* and *TBX*15^21^.

Differential expression of developmental genes, including *HOX* and *TBX* family members, between omental VAT and abdominal SAT, and between different SAT depots has previously been reported^31^. Our data highlight that many TF genes with important developmental roles in adipogenesis and more broadly in regionalisation (Fig. 3d) are both differentially expressed and are marked by distinct DNA promoter methylation profiles in SA and VA. Indeed, this set of genes includes many of those with the largest DMRs (Supplementary Fig. S8). The likelihood that TF genes are differentially expressed between SA and VA increases with the strength of the DMR with 16/23 (70%) genes with DMRs >0.4 being differentially expressed (Supplementary Fig. S8, χ^2^ trend, 67.63, p = 1.374e-14). For example *WT1*, expression of which has been associated with visceral adipocyte development in mice^30^, is more highly expressed in VA relative to SA and is marked by differential methylation of >30%. The promoters of the developmental genes *BARX1*, *EBF2*, *NKX3-2* and *GATA2* are also marked by promoter DMRs with ΔMe ≥0.3, Supplementary Table S13. Among other genes that are marked by differential promoter methylation and are more highly expressed in VA (Fig. 3d) are engrailed (*EN1*), *HOXB6* and the key adipocyte transcription factor *ZNF423*. In contrast to visceral depots, the developmental origins of subcutaneous depots remain largely unknown^30^. In SA we see numerous developmental transcription factors that are epigenetically marked and overexpressed relative to VA. These include multiple members of the *HOX* gene families (including *HOXA9*, *A10* and *A11*, *HOXC6* (previously described^15^), *C8* and *C10, HOXD3* and *D8*), *TBX* genes (*TBX4*, *15* and 18) as well as *IRX3*, *PITX2*, *ALX1* and *DMRT3*. These genes provide a gene expression and DNA methylation signature that is indicative of the different development origins of SA and VA.

Elevated DNA methylation in promoter regions is commonly associated with reduced gene expression. Most DE-DM genes (62%) showed the expected inverse relationship between gene expression and promoter methylation. However, surprisingly, for the remaining 38% of genes, differences in gene expression and promoter methylation were concordant, that is elevated methylation within 2 kb of the TSS was associated with increased gene expression. We found that the concordant genes were significantly enriched for TFs and commonly located in DMVs (Supplementary Fig. S8). Examples (blue shade) are shown in Fig. 3e,f (*GATA2* and *HOXC10* and Supplementary Fig. S9 (*ALX1*, *DMRT3*, *EN1*, *IRX3*, *TBX18*, and *SLFN12L*). In these examples the differential methylation shortens the unmethylated region surrounding the TSS in the higher-expressing cell type.

The differences in DNA methylation at these transcription factor promoters are often of high magnitude and are characteristically in DMVs suggesting that DNA methylation plays a key regulatory role in defining fat depots during development.

### Identification of regulatory elements from methylome profiles

**Figure 4:**
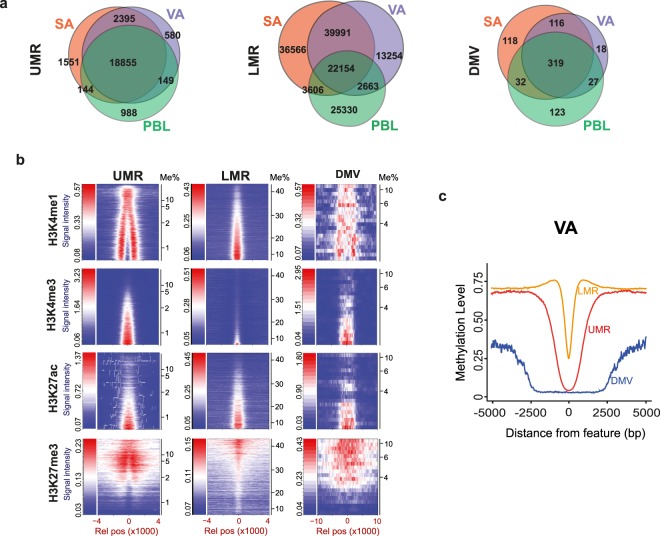
DNA methylation and regulatory elements. **(a)** Venn diagrams showing number of shared and tissue specific UMRs, LMRs, DMVs across SA, VA and PBL. **(b)** Signal intensity plots of subcutaneous adipocyte H3K4me1, H3K4me3, H3K27ac and H3K27me3 levels (from The Roadmap Epigenetics Project) across SA UMR, LMR and DMV features relative to the centre of each feature. Individual regions (rows) are stacked from the lowest to highest mean methylation across the regions. Signal intensity is a gradient of blue at lowest intensity and red at highest. **(c)** Smoothed averages of VA methylation levels at LMRs (yellow), UMRs (red) and DMVs (blue). Plots are centred at the midpoints of each feature.

We applied MethylSeekR^32^ to the DNA methylation profiles of VA, SA and PBL samples to identify putative regulatory elements, as characterised by methylation levels and CpG site density. Unmethlyated regions (UMRs) have previously been reported to represent active promoters and low-methylated regions (LMRs) to represent active enhancers^33^ (Supplementary Tables S15–20). Additionally, we identified extended regions of minimal DNA methylation (DMVs) commonly associated with genes regulating early development^34^ (Supplementary Information, Supplementary Table S21). Venn diagrams (Fig. 4a) show the overlaps of UMRs, LMRs and DMVs identified in SA, VA and PBL cells. The predictive classifications of these elements were validated using available (NIH Roadmap Epigenomics Project) chromatin data of subcutaneous adipocytes (Fig. 4b).

Of the 24,662 UMRs identified, 18,855 (76%) were shared between the VA, SA and PBL DNA (Fig. 4a, Supplementary Fig. S10). A further 2,395 (9.7%) were shared between the two adipocyte types alone while only 12.6% were specific to an individual cell type, including 580 VA specific and 1551 SA specific UMRs. This substantiates that the majority of UMRs are not tissue specific and are unmethylated independent of expression of the associated gene. UMRs typically encompass regions spanning ~1000 bp (Fig. 4c and Supplementary Fig. S10) and are found near TSSs (Supplementary Fig. S10), with >70% of UMRs overlapping a TSS (Supplementary Tables S15–17). We found that SA UMRs correlate strongly with the active promoter marks (H3K4me3 and H3K27ac) and enhancer marks (H3K27ac and H3K4me1) (Fig. 4b). Interestingly H3K4me1 displays a ‘wishbone’ distribution bordering the H3K4me3 mark and its binding is enriched at UMRs with a lower density of DNA methylation (Fig. 4b).

LMRs are 3 to 6 times more frequent than UMRs, in all tissues (Supplementary Fig. S10) and notably are more tissue specific, with less than 16% (22,154) of 143,464 identified LMRs shared between PBL, SA and VA samples and 28% (3,991) between SA and VA samples (Fig. 4a), supporting the overall tissue specificity of enhancer elements. LMRs are generally shorter than UMRs, a few hundred bp (Fig. 4c and Supplementary Fig. S10), are further from the nearest TSS, with a median distance of ~10 kb (Supplementary Fig. S10) and are characterised by enrichment for enhancer marks H3K4me1 and H3K27ac and deplete of H3K4me3 and the repressive mark H3K27me3 (Fig. 4b). To assess the utility of UMRs and LMRs for identifying regulatory elements in SA and VA, we performed GO analysis comparing adipocyte-specific elements (i.e. those common to SA and VA, but not PBL) with PBL-specific elements. Tissue-specific UMRs were found to be enriched in terms associated with the difference in cell type. For example, adipocyte-specific UMRs were enriched for lipid related terms including triglyceride biosynthetic process (Supplementary Fig. S11, Supplementary Table S22), while PBL-specific UMRs were enriched for blood and immune terms such as leukocyte migration (Supplementary Fig. S11). In contrast, tissue specific LMRs showed a wide range of enriched terms including those relating to extracellular matrix, blood vessel morphogenesis and developmental processes, supporting their role as regulatory elements in a wide range of pathways important in adipocyte biology (representative example, SA LMRs Supplementary Fig. S11, Supplementary Information, Supplementary Table S22).

DMVs were found at lower frequencies ~500 to 600 in each sample (Supplementary Fig. S10). The least methylated DMVs were associated centrally with the active marks H3K27ac and H3K4me3. In contrast the modest increases in methylation levels (4–10%) were accompanied by an increased enrichment of the repressive H3K27me3 mark (Fig. 4b). Consistent with Xie and colleagues^34^, genes associated with DMVs were highly enriched for transcription and developmental processes (Supplementary Fig. S11, Supplementary Table S22).

### Preferential association of transcription factor binding sites with regulatory regions inferred from DNA methylation profiles

**Figure 5:**
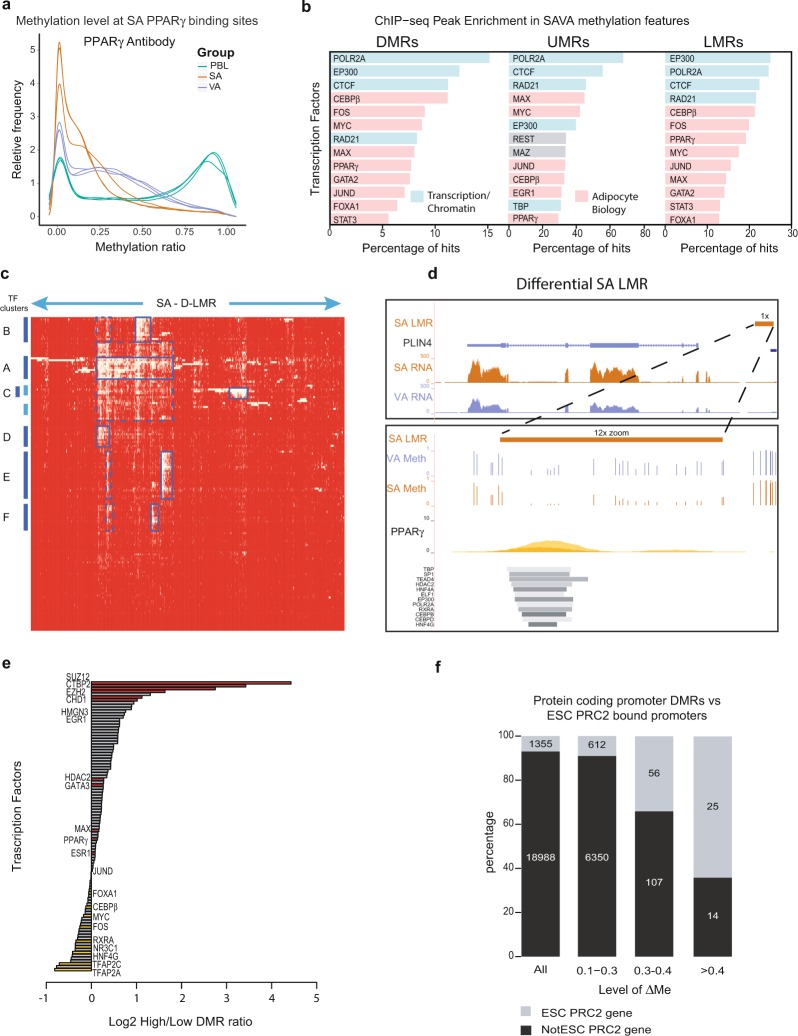
Association of Transcription Factor Binding Sites with DNA Methylation Features. **(a)** Density plot of the relative frequency of SA PPARγ ChIP-seq peaks, y-axis^35^, against the level DNA methylation within these regions (x-axis) determined from our SA, VA, PBL WGBS data. **(b)** Plots of frequencies of the presence of transcription factor (TF) ChIP-seq peaks within DNA methylation features: DMRs (left panel), and UMRs (centre panel) and LMRs (right panel) in SA and VA, but not PBL. Blue bars are TFs involved in general transcriptional regulation or chromatin architecture, Pink bars are TFs involved in adipocyte biology, and grey bars are TFs involved in neither. **(c)** Unsupervised cluster plot of the presence (yellow) or absence (red) of ChIP-seq peak for 162 TFs (vertical axis) within 4,986 SA D-LMRs (horizontal axis). TF clusters A-F represent clusters of regions containing binding sites for common set of TFs. **(d)** UCSC browser image of *PLIN4*. Upper panel = broad view (1x zoom): SA D-LMR (orange), SA RNA-seq (orange), VA RNA-seq (purple). Lower panel = zoomed in view (12x zoom) of SA D-LMR (orange) and WGBS methylation tracks: VA Meth (purple) and SA Meth (orange) as well as PPARγ ChIP-seq (yellow and orange)^35^ and various transcription factor ChIP-seq peaks (grey bars)^37^. **(e)** Plot comparing the percentage of High (ΔMe ≥ 0.3) and Low (ΔMe 0.1 to <0.3) DMRs that contain particular TF ChIP-seq peaks [ENCODE^35^]. A ratio was generated with the percentage of High DMRs containing ChIP-seq peaks over the percentage of Low DMRs for each TF. This ratio was plotted on a log2 scale – x-axis. Selected TFs are highlighted in text and by red (positive ratio) and yellow (negative ratio) bars. **(f)** Barplot of protein coding genes stratified into, all protein coding promoters (All), and those with promoter DMRs between 0.1 to <0.3, 0.3 to <0.4, and ≥0.4. The percentage of these genes are then split by whether their promoters are bound by PRC2 in ES cells (grey)^37^ or are not bound by PRC2 in ES cells (black). Y-axis = percentage of promoters that contain ESC PRC2 ChIP-seq peaks or not, x – axis = gene stratification.

Transcription factors can associate combinatorially with gene regulatory elements to drive cell type-specific gene expression. To investigate differences in the transcriptional landscape between SA and VA we overlapped publicly available TF ChIP-seq data with the potential regulatory elements we had identified from WGBS. We initially used binding sites for the key adipocyte transcription factors PPARγ and CEBPα that have previously been mapped using ChIP-seq in subcutaneous adipocytes derived by differentiation of primary human pre-adipocytes^35^ or the established SGBS human cell line model, Schmidt, *et al*.^36^. We found that PPARγ binding regions identified in the primary subcutaneous adipocyte model^35^ were strongly enriched for low levels of methylation in SA (Fig. 5a, x-axis). In contrast, the same PPARγ sites showed intermediate levels of methylation in VA samples and mostly high methylation in PBL DNA. SGBS cells showed a similar trend of enrichment, with binding sites for PPARγ and CEBPα overlapping regions of low SA methylation (Supplementary Fig. S12). These data highlight the relationship between transcription factor binding and DNA methylation and reveal differences in DNA methylation between SA, VA and PBL at binding sites for the adipogenesis regulatory TFs PPARγ and CEBPα.

Looking more broadly at the binding of other TFs we utilised the set of ChIP-characterised binding sites for 161 factors curated by the ENCODE Consortium^37^, across many different cell types. For each TF we mapped the presence of ChIP-determined binding sites within UMRs and LMRs common to SA and VA, but not PBL, and SA/VA DMRs that distinguish differences in methylation between VA and SA. Across all methylation features, binding sites were highly enriched both for general transcription proteins such as POLR2A and proteins associated with chromatin architecture (EP300, CTCF, RAD21), as well as sequence-specific TFs (Fig. 5b, Supplementary Table S23). Indeed approximately 90% of tested TFs showed high level enrichment with the features (p < 0.001, hypergometric test, Supplementary Table S23). Within the set of SA/VA DMRs, all nine sequence-specific TFs with binding sites in ≥5% of DMRs, including CEBPβ (11.2%), FOS (9.0%) and PPARγ (7.7%), are of known importance in adipocyte biology (Fig. 5b).

Comparison of TF binding within LMRs and UMRs that were shared by SA and VA and not PBL, with those found only in PBL showed that these shared UMRs and LMRs were enriched in binding sites of known TFs important in adipocyte biology such as PPARγ (Fig. 5b, Supplementary Tables S23 and S24). The *ZNF423* and *CEBPA* genes, important regulators of adipogenesis, harbour adipocyte-specific UMRs and LMRs respectively, which are bound by multiple TFs (Supplementary Figs S12, S13).

To explore whether LMRs could be used to identify putative, functionally important, enhancer elements that were specific to each cell type, we investigated the relationship between differentially expressed genes and TF binding in LMRs specific to SA or VA. We separately chose SA and VA LMRs with the lowest 25% of methylation (these LMRs displayed the highest enrichment of active histone marks in SA – Fig. 4b) and overlapped these with VA- or SA-DMRs respectively. This process identified potential enhancer elements showing distinct differences in DNA methylation between SA and VA, which we termed SA D-LMRs (4,986) and VA D-LMRs (862) (Supplementary Table S25). Of the 3,455 genes that are the nearest protein coding genes to these D-LMRs, ~22% were differentially expressed compared with 13% of all protein-coding genes (χ^2^ = 176.5, two-tailed *p* < 2.2e-16). By overlapping D-LMRs with ChIP-seq binding peaks (Supplementary Tables S26, S27) we found similar enrichment levels for many of the same TFs in both SA and VA D-LMRs (Supplementary Fig. S13). This suggests that, in many instances, the same key DNA binding factors utilise different enhancer elements in SA and VA. Unsupervised clustering of SA D-LMRs and TF binding sites (Fig. 5c) shows clustering of groups of TFs associated with subsets of the LMRs. A similar pattern of clustering is seen for VA D-LMRs (Supplementary Fig. S14). The most prominent cluster (A) identifies LMRs that contain binding sites for the adipocyte TFs PPARγ and CEBPβ, STAT3, as well as a set of common transcriptional regulators and chromatin modifiers, including EP300, POL2RA, MAX, MYC and FOS/JUN family members. Additional clusters identify sets of TFs that might also commonly co-operate in gene regulation (discussed in Supplementary Information). For example, cluster F includes the TF NR2F2 that has been shown to be a critical regulator of adipogenesis^38^. More than 50% of SA D-LMRs containing binding sites for NR2F2 also contain binding sites for TEAD4, GATA2, TAL1, CEBPβ and PPARγ These enriched clusters of TF binding sites are consistent with the LMRs having transcriptional regulatory roles in adipocyte differentiation and function.

To locate potential regulatory regions distinguishing SA and VA we identified D-LMRs associated with multiple TFs and overlapped these with genes differentially expressed in VA and SA. This process identified LMRs potentially regulating expression of specific genes involved in adipocyte function (Supplementary Information) including *F13A1*, the Wnt signaling receptor *LRP5* (Supplementary Fig. S14)*, SLC2A5*, *CIDEC* and *TPCN2*. The example of *PLIN4* (LMR in Cluster B) a protein essential to lipid droplet formation in adipocytes^39^ is shown in Fig. 5d. Together these examples point to the potential to utilise DNA methylation profiles combined with TF binding data to identify candidate genomic regions involved in regulation of adipocyte gene expression.

### The most differentially methylated regions overlap with Polycomb Repressive Complex 2 binding regions

Given the strong association of High SA/VA DMRs, with developmental regulators, in contrast to Low DMRs, we next investigated whether they might be preferentially bound by different transcription factors. Consistent with the association of Low DMRs with genes involved in metabolic/cellular functions, binding sites for TFs such as RXRα (PPARγ binding partner), the glucocorticoid receptor (NR3C1), and TFAP2 family members are among those showing modest enrichment (Fig. 5e – negative values, Supplementary Table S28). For both SA and VA, High DMRs were strongly enriched in binding sites for SUZ12 and EZH2 (Fig. 5e), core subunits of the Polycomb Repressive Complex 2 (PRC2), an important regulator in early development^10^. Interestingly, CTBP2, which is involved in early lineage commitment through recruitment of the NuRD and PRC2 complexes^40^ and plays a direct role in adipogenesis^41,42^, was also highly enriched.

To further explore the relationship between High DMRs and developmental pathways we investigated public data of PRC2 binding sites (EZH2/SUZ12 peaks) in embryonic stem cells (ESCs)^37^. We overlapped SA/VA promoter DMRs with EZH2/SUZ12 peaks from ESCs and found that the higher the ΔMe the more likely the regions were to be bound by EZH2/SUZ12 in ES cells. Of the protein coding genes with promoter associated Low DMRs only 9% had promoters bound by EZH2/SUZ12 in ESC, in contrast to 34% of protein coding genes with High DMRs (Fig. 5f) (χ^2^ 216.7, p < 2.2e-16). As genes with High DMR promoters were also frequently differentially expressed, these findings highlight how, through DNA methylation, events that occur in early development may later influence gene expression and cell function in mature adipocytes.

## Discussion

To further our understanding of the molecular differences between subcutaneous and visceral adipocytes, we have developed the first comprehensive DNA methylome and transcriptome maps of adipocytes isolated from healthy weight individuals. The gene expression data highlights that many of the functional differences seen in transcriptome analysis of adipose tissue are substantially contributed to by the adipocytes themselves, for example in the processes of angiogenesis, extracellular matrix organisation and inflammatory responses. DNA methylation profiles were notably distinct between the different adipocyte depots. The most striking differential methylation that separated VA and SA was found at transcription factor and developmental genes and these regions were subject to polycomb repression early in development. These findings highlight that early cell fate decisions and different developmental origins are reflected in the differential DNA methylation landscapes of SA and VA.

Analysis of differential methylation identified a clear difference between High (ΔMe ≥ 0.3) and Low (ΔMe, 0.1–0.3) DMRs. High DMRs were positively associated with developmental TFs, while low DMRs were enriched for pathways related to cellular metabolism, extracellular matrix and responses to external signals. Likewise, many genes that had promoter DMRs and were differentially expressed have recognised roles in the biology of fat tissue, indicating the importance of DNA methylation in shaping the differential biological properties of adipose depots. DNA methylation profiles across both VA and SA were used to identify potential active promoters and enhancers (UMRs and LMRs respectively). The clear clustering of groups of transcription factors on subsets of LMRs and UMRs is indicative of specific combinations of TFs co-operating in adipocyte gene regulation. Combining gene expression data with specific regulatory (UMRs or LMRs) elements that are shared (eg., *CEBPA* gene) or differ (eg *LRP5* gene) between SA and VA provides a pathway to use this catalogue of cis-regulatory elements for identification and study of putative regulatory regions controlling differential gene function in adipocytes.

An unexpected feature observed at a number of genes with higher relative differential expression, was the presence of hypermethylated promoter DMRs. Many of these “same direction” DE-DM genes (eg. *HOXC10* and *GATA2*) demonstrated a sharp delineation of the DNA methylation profile at the active promoter region; we coined these DMRs methylation ‘cliffs’. A similar feature of differential methylation bordering and potentially defining promoter boundaries has been seen in the differentiation of hematopoetic stem cells to mature cells of the myeloid and lymphoid lineages^43^. Recent work from Li *et al*.^44^ also identified a similar relationship in mouse tissues where they found a subset of dynamic DMVs that exhibited a positive correlation between DNA methylation and gene expression. Likewise, they found that these genes were enriched for TFs. Based on the observation of these “same direction” DE-DM genes we speculate that, when broadly unmethylated, these regions are associated with repressive chromatin marks (PRC2-dependent), and that modelling of the active state involves removal of repressive chromatin that covers the active promoter. This is accompanied by methylation of the cliff regions that flank the core active promoter, thus limiting the encroachment of repressive chromatin.

Relative to other cell types in the body SA and VA are morphologically and functionally very similar, providing an ideal model to examine how the epigenome helps to define cell type. As mentioned above we find that the transcriptional differences between SA and VA match well with known differences in functional pathways. However, the most striking contrast is in their differential methylation where there is a clear enrichment of developmental genes, particularly for High DMRs. Previous studies have demonstrated that a cell’s DNA methylation profile provides an epigenetic memory of its developmental lineage^45–47^. Unlike many of the organs and tissues that arise from the mesoderm, such as kidney, gonad and limb, the process of development of the adipose depots is poorly described. Interestingly we find here that many of the differentially expressed and methylated TFs are genes important in the developmental processes of regionalization and positional identity. Differential expression and methylation of the *PITX*, *HOX* and *TBX* families, suggests that, like in the regionalization processes for other tissues, these genes may play a role in defining visceral from subcutaneous depots. Developmental genes, including *HOX* and *TBX* family members, have been shown at the tissue level to be differentially expressed across different fat depots including between omental VAT and abdominal SAT^31^. Our data on isolated SA and VA significantly extends the suite of developmental genes whose expression distinguishes abdominal subcutaneous and visceral tissues and demonstrates that many of these genes carry distinct epigenetic marks in their promoters. Indeed, this set of genes includes many of those with the largest DMRs. The promoters of TFs enriched for high magnitude DMRs were also enriched for PRC2 binding in ESCs. This further supports the role of these genes as lineage differentiation factors^10^, as genes repressed by PRC2 in early development and very likely regulated by promoter DNA methylation in mature adipocytes. The strong enrichment for promoter High DMRs at TFs that are differentially expressed, suggests that these regions are not simply residual markings left over from developmental history, but that they are important in regulating transcriptional activity in mature adipocytes. These data also support the idea that DNA methylation may play a particularly important role in regulating TFs, compared to other genes.

Lineage tracing experiments in mice have shown that visceral, in contrast to subcutaneous, adipocytes are derived from Wt1-expressing cells of the lateral plate mesoderm^30^. Elevated relative expression of *WT1* in VA and differential promoter methylation of >30% also supports that the lateral plate mesoderm is a source of visceral adipocytes in humans. In contrast to visceral depots, the developmental origins of subcutaneous depots remain largely unknown^30^. Here we see numerous developmental transcription factors that are epigenetically marked and overexpressed in SA relative to VA. These genes, including *HOXC10*, *TBX4* and *PITX2*, provide a gene expression and DNA methylation signature indicative of the different development origins of SA and VA. Interestingly in mice, *Tbx4/5*, *Pitx1/2* and numerous *Hox* genes (including *HoxC9/10*) are expressed in the lateral plate mesoderm^48,49^ and regulate limb development. The fore- and hindlimbs have also been shown to arise from different sub-domains of the lateral plate mesoderm and their development is differentially regulated by key TF genes *Tbx4* and *HoxC10* (hind-limbs) and *Tbx5* (fore-limbs)^49^. Similarly, we hypothesize that progenitors from different mesoderm sub-domains give rise to the origin of VA and SA, and this is dependent on whether the cells either express *WT1* (VA) and different combinations of *TBX, HOX* and *PITX* genes. Recently, HOXC10, TBX4/5 and PITX1 proteins have been shown to cooperate in development of mouse limb buds, with HOXC10 and TBX4 interacting directly to control a common set of downstream genes^50^. This raises the possibility that HOXC10 interacting with TBX4, along with PITX2 (all expressed in SA) may also cooperate in driving development of the subcutaneous pre-adipocyte lineage. The differential role of *TBX5* gene expression in abdominal and gluteal SAT depots^51^ exemplifies the significance of such lineage differentiation factors. *TBX5* is both differentially methylated and expressed between the depots, and knockdown of *TBX5* expression affected proliferation and differentiation of abdominal SAT preadipocytes.

Overall, these findings highlight that early cell fate decisions are reflected in the methylomes of SA and VA, with major differences in methylation strongly enriched around genes important in development. These data support the hypothesis that early developmental events are important in defining the transcriptional profiles of SA and VA, and that these differences are established and maintained, at least in part, by DNA methylation. This concept is likely to be common to other cell types and demonstrates that WGBS mapping provides a powerful approach to interrogate gene regulation and lineage history.

## Methods

### Subjects and tissue samples

Three normal weight female subjects undergoing elective surgery were recruited under the study protocol approved by the St Vincent’s Hospital Human Research and Ethics Committee, SVH File Numbers: H06/151 and 12/200. All subjects gave informed consent for participation. All experiments, data collection and storage conformed to the approved study protocol, and in accordance with relevant guidelines and regulations. The women were aged 35–47, with BMIs ranging from 19.1 to 25.4. Further details on subjects is available in Supplementary Information and Supplementary Table S1.

VAT was collected from the greater omental region and SAT from the periumbilical site of surgical incision. Details of methods for tissue processing, adipocyte isolation and nucleic acid isolation are provided in Supplementary Information.

### DNA Methylome analysis

Whole genome bisulfite sequencing libraries were prepared from 1 µg of DNA following Illumina’s “Whole- Genome Bisulfite sequencing for Methylation Analysis” protocol. Three lanes of paired end 100 bp sequencing was performed for each of the library on the Illumina HiSeq2500 platform using the TruSeq v3 cluster kits and SBS kits to achieve coverage ranging between 25–30x. (details in Supplementary Table S1). Supporting DNA methylome data on the core and two healthy weight male subjects was obtained using Illumina Infinium Bead Chip Human450 arrays. Further experimental details, details of quality assessment and sequence alignment, and analysis of differential methylation and methylation profiles are provided in Supplementary Information and Supplementary Table S1.

### Gene expression

Libraries for Poly-A and Whole RNA-seq were prepared from 500 ng input RNA using the Illumina TruSeq Stranded poly-A RNA Library Prep Kit RNA-seq and following ribosomal RNA reduction using Illumina TruSeq Stranded Total RNA Library Prep Kit respectively. Poly-A RNA-seq libraries were sequenced using 100 bp paired-end HiSeq2500 chemistry3 yielding at least 30 million reads per sample, Supplementary Table S1. Whole RNA-seq libraries were sequenced using 100 bp paired-end HiSeq2500 chemistry4 yielding 35 to 40 million aligned reads per sample (see Supplementary Information and Supplementary Table S1). Details of quality checking, mapping of reads to the genome, and to genes/transcripts, calling of differential gene/transcript expression and Gene Ontology analysis are provided in Supplementary Information.

### Data access

Whole genome bisulfite sequencing data, RNA sequencing data and DNA methylation array data from this study are available from the NCBI’s Gene Expression Omnibus repository (GEO; http://www.ncbi.nlm.nih.gov/geo/) under the series accession number GSE110821.

## Supplementary information

Supplementary Information

Supplementary_Table S1

Supplementary_Table S2

Supplementary_Table S3

Supplementary_Table S4

Supplementary_Table S5

Supplementary_Table S6

Supplementary_Table S7

Supplementary_Table S8

Supplementary_Table S9

Supplementary_Table S10

Supplementary_Table S11

Supplementary_Table S12

Supplementary_Table S13

Supplementary_Table S14

Supplementary_Table S15

Supplementary_Table S16

Supplementary_Table S17

Supplementary_Table S18

Supplementary_Table S19

Supplementary_Table S20

Supplementary_Table S21

Supplementary_Table S22

Supplementary_Table S23

Supplementary_Table S24

Supplementary_Table S25

Supplementary_Table S26

Supplementary_Table S27

Supplementary_Table S28
