# Supplementary material for: Methylome and transcriptome maps of human visceral and subcutaneous adipocytes reveal key epigenetic differences at developmental genes
Source: Sci Rep. 2019 Jul 2;9:9511. doi: 10.1038/s41598-019-45777-w (PMC6606599; doi:10.1038/s41598-019-45777-w)
Supplement: Supplementary file 1 — Supplementary Information [file 41598_2019_45777_MOESM1_ESM.pdf]

Methylome and transcriptome maps of human visceral and subcutaneous adipocytes reveal key epigenetic differences at developmental genes.

Authors: Stephen T. Bradford, Shalima S. Nair, Aaron L. Statham, Susan J. van Dijk, Timothy J. Peters, Firoz Anwar, Hugh J. French, Julius Z.H. von Martels, Brodie Sutcliffe, Madhavi P. Maddugoda, Michelle Peranec, Hilal Varinli, Rosanna Arnoldy, Michael Buckley, Jason P. Ross, Elena Zotenko, Jenny Z. Song, Clare Stirzaker, Denis C. Bauer, Wenjia Qu, Michael M. Swarbrick, Helen L. Lutgers, Reginald V. Lord, Katherine Samaras, Peter L. Molloy, Susan J. Clark

SUPPLEMENTARY INFORMATION

Supplementary Results and Discussion	**Page**
Differential gene expression between SA and VA	**2**
Gene expression in VA compared with VAT	**2**
Supplementary Fig.S1	**3**
DNA methylation analyses	**4**
Supplementary Fig.S2	**4**
Differentially Methylated Regions<br>Supplementary Fig.S3	**5**
Association of DMRs with body fat distribution and obesity-related SNPs	**6**
Ontology of genes associated with DMRs	**8**
Supplementary Fig. S4	**8**
DMRs associated with genes encoding Transcription Factors	**9**
Supplementary Fig. S5	**9**
Supplementary Fig. S6	**10**
Relationship of DNA methylation and DMRs with gene expression	**11**
Supplementary Figs. S7-S9	**11-13**
Regulatory elements identified within DNA methylomes of purified SA and VA	**15**
Supplementary Figs. S10-S13	**14-17**
Clustering of TF binding sites in D-LMRs<br>Supplementary Fig. S14	**18**
Materials and Methods	**22**
List of Supplementary Tables	**28**
Glossary for Supplementary Tables	**28**
Supplementary References	**31**

Supplementary Results and Discussion

Differential gene expression between SA and VA

The transcriptomes of SA and VA were generated, using both polyA and whole transcriptome RNA-seq and aligned against a set of 57,818 genes. Differentially-expressed (DE) genes were determined in each dataset, **(Supplementary Tables S20 and S21 respectively**). Levels of differential expression were highly concordant, R^2=0.9144 (**Supplementary Fig. S1a**), so a combined set of DE genes was used for subsequent analysis.

As well as genes discussed in the main text, a number of genes that were highly differentially-expressed warrant consideration in relation to the differential biology of SA and VA. Those most differentially expressed in VA include:

- cell surface proteins mesothelin (*MSLN*), uroplakin 3B (*UPK3B*) and mucin16 (*MUC16*),
- cytokeratin 19 (*KRT19*)
- LDL receptor related protein 2 (*LRP2*) that binds a variety of ligands, including lipoprotein lipase and has been shown to be involved in leptin signalling (Byun et al. 2014)
- *PKHD1L1*, potentially associated with immune function (Hogan et al. 2003) and
- arachidonate lipoxygenase 15 (*ALOX15*) for which linoleic acid also a substrate (Cole et al. 2012).
- *ISL1*, that has been shown to be a key early driver of adipogenesis in 3T3-L cells (Ma et al. 2014)
- Basonuclin (*BNC1*) a zinc finger transcription not previously associated with a function in adipose tissue.

The most highly differentially-expressed gene in SA is *SIM1* (Drosophila single-minded (sim) gene homolog) that has been associated with obesity, but where studies to date have been focused toward neural effects and hyperphagia (Xi et al. 2012). Other genes much more highly expressed in SA are Tubulin-beta-2A (*TUBB2A*), the homeobox transcription factor *IRX5* and the divergently-transcribed lncRNA *CRNDE* that is involved in insulin signalling (Ellis et al. 2014), Cyclin D1, *CCND1*, required for pre-adipocyte proliferation (Marquez et al. 2017), and its neighbour *MYEOV*.

Notably most of these genes that show highly differential expression between isolated visceral and subcutaneous adipocytes have been shown to be differentially expressed in studies of whole tissue, significant overlap (Fisher pval = 4.41e-07) was found between our list of differentially expressed genes and those identified by Wolfs and colleagues in a study examining whole tissue SAT and VAT from 75 obese adults (Wolfs et al. 2010). Of the 810 genes we found to be up in SA 275 of these were also found to be up in SAT vs VAT, while 333 of the 1395 genes down regulated in SA were also found to be down between SAT and VAT in their data.

Gene expression in VA compared with VAT

Comparing the expression profiles of VAT with isolated VA we see, as expected, that expression in whole adipose tissue is quite distinct from the isolated adipocytes. In VAT we see higher expression of genes expressed in certain non-adipocyte cell types such as *CSF1R* (macrophage/monocyte), *CD34* (blood) and *MMRN1* (endothelium) (**Supplementary Table S5**). GOseq analysis of VAT-

enriched genes also highlights non-adipocyte pathways with a strong enrichment for immune system terms (immune system process FDR 1.81e-53) (**Supplementary Table S6**).

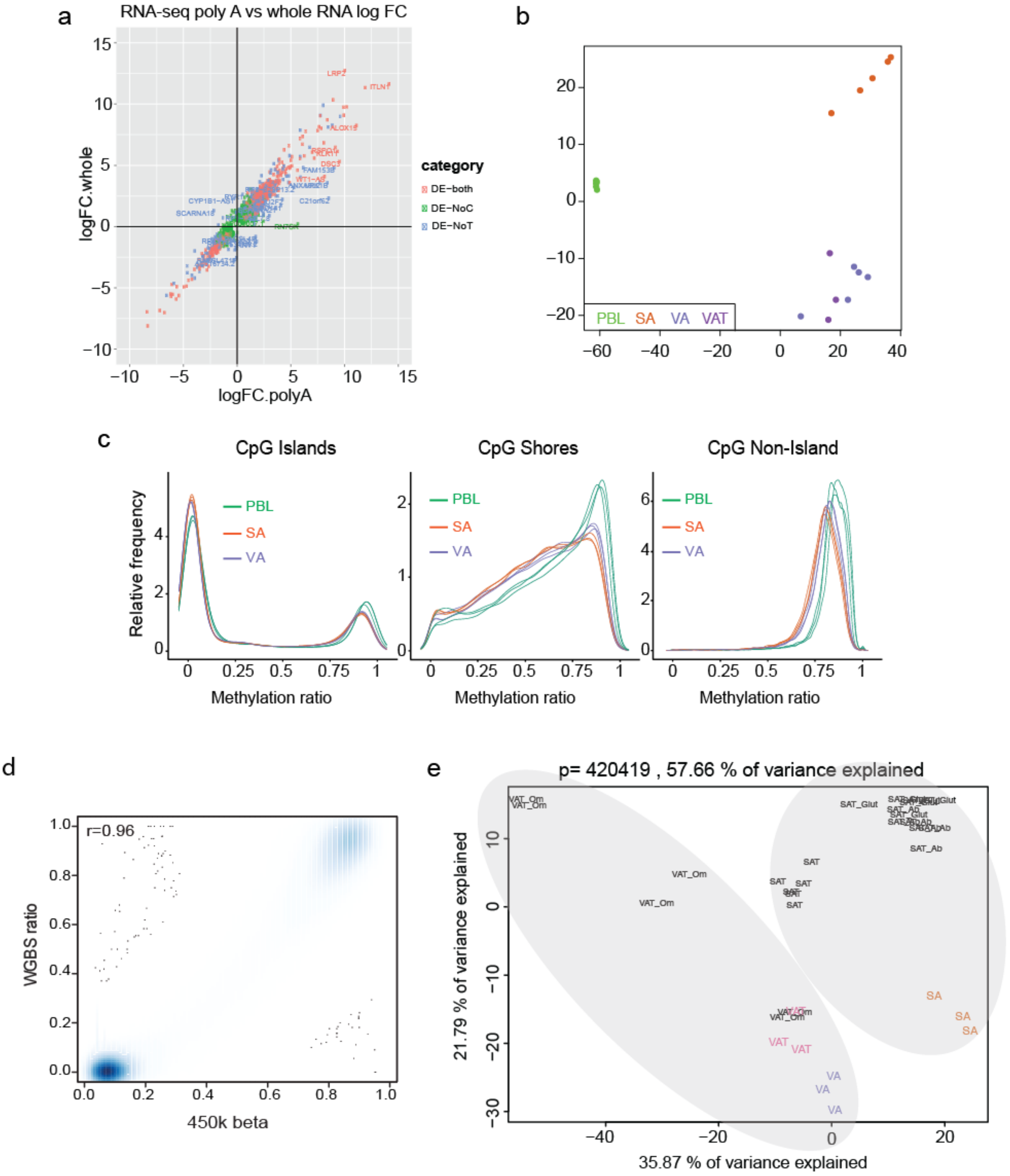

Supplementary Figure S1. Correlation and clustering of transcriptome and methylation data.

(a) Plot of correlation of PolyA RNA-seq vs whole RNA-seq based on logFC. DE-both (red) = DE in both RNA-seq, DE-NoC (green) = DE in polyA only, DE-NoT (blue) = DE in whole only. **(b)** MDS of 450k samples based on methylation at 483,765 probes **(c)** Smoothed density plots at single CpG resolution of methylation level across CpG Islands, CpG shores and CpGs in neither islands nor shores **(d)** Correlation of 450k beta values with WGBS methylation fraction at CpGs sites present on 450k array for a representative VA sample **(e)** MDS of 450k samples from VA, SA and VAT along with publically available VAT and SAT data (Gehrke et al. 2013; Slieker et al. 2013); p = 420,419 with 57.66% of variance explained.

DNA methylation analyses

As well as WGBS of the core three subjects, the same DNA samples, as well as samples from two normal weight male subjects (**Supplementary Table S1**) were analysed using Illumina Infinium Bead Chip Human450 arrays (450K arrays). The MDS plot of 450K array data for the 5 individuals showed very similar clustering as WGBS data (**Supplementary Fig. S1b**), and for CpG sites on the 450K arrays, WGBS and 450K data were highly correlated, with a Pearson's correlation coefficient >0.94 for all sample comparisons (**Supplementary Fig. S1d**) and. Comparison of 450K data from our samples with publically available data on SAT and VAT showed a broad clustering by depot type (**Supplementary Fig. S1e**)

450K data was used to examine variance of methylation levels at each CpG site. Median variance was about 1.5% (**Supplementary Fig. S**2a). Lowest variance was found promoter and first exon regions, corresponding with regions of very low methylation including CpG islands. Higher variance was seen at 3' UTRs, intergenic and gene body regions (**Supplementary Fig. S**2b). Variance at individual CpG sites was well correlated between SA and VA. The minority of CpG sites that show high variance in methylation commonly show such variance in SA, VA and PBL of the same individual, suggesting a genotype influence rather than being tissue-specific effects (**Supplementary Fig. S2c, d**).

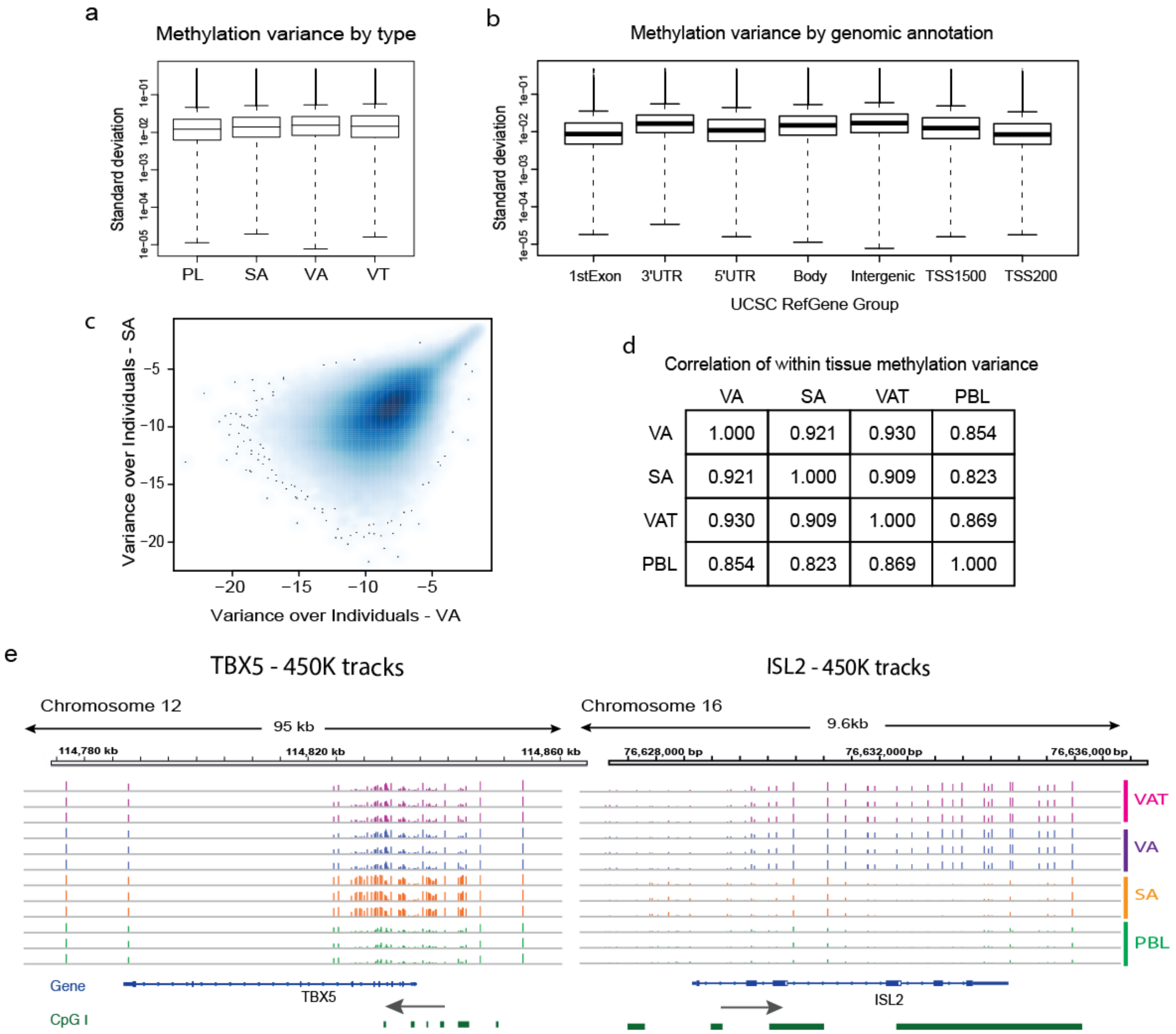

Supplementary Figure S2. Concordance of DNA methylation between individuals.

(a) Box plots of the within tissue variance of DNA methylation for SA, VA, VAT and PBL, as measured across all probes on 450k arrays. Y-axis = standard deviation, x-axis = annotation category. **(b)** Box plots of the variance of DNA methylation of combined tissues across genomic annotations as measured by 450k array. Y-axis = standard deviation, x-axis = annotation category. **(c)** Plot of correlation of within tissue variance of DNA methylation as measured by 450k array between SA and VA. **(d)** Table of correlation of within tissue variance of DNA methylation as measured by 450k array, for all tissue combinations. **(e)** Browser shots of 450k methylation levels at individual CpG sites across *TBX5* and *ISL2* loci respectively, for VAT, VA, SA and PBL from individual patient samples, green bars indicate CpG Islands.

Differentially Methylated Regions

Comparing VA and SA 450K array data for the 5 subjects we identified 2,393 DMRs (1,493 up in SA and 900 down in SA) (**Supplementary Table S9**). We found significant overlap in differential methylation between our 450K and WGBS data. Using GenomicRanges (Lawrence et al. 2013) to directly determine overlaps of DMRs, 1,728 450K DMRs (72%) overlapped or were contained within 1,910 WGBS DMRs (**Supplementary Fig. S3d**). A strong overlap of differential methylation is also evident at the single CpG level. Using the normally distributed test statistics for differential methylation, we tested concordance of the differential signal between 450K and WGBS, for CpGs with probes on the 450K array. There was an overall concordance value of r = 0.63 (**Supplementary Fig. S3e**).

Recently, Macartney-Coxson et al. (Macartney-Coxson et al. 2017) used 450K arrays to compare the DNA methylomes of subcutaneous and visceral adipose tissue of 15 morbidly obese subjects before and after weight reduction following bariatric surgery. They identified 703 DMRs associated with 385 genes in common between before and after surgery SA/VA comparisons. We used GenomicRanges to identify overlaps between our 450K DMRs identified on isolated SA and VA and these 703 DMRs. 438 (62%) of the DMRs were shared with ours. This strong overlap is despite our comparison being of isolated adipocytes of normal weight subjects and theirs of whole adipose tissue from obese subjects. Notably, highly differential methylation in regions of important developmental genes was observed in both datasets.

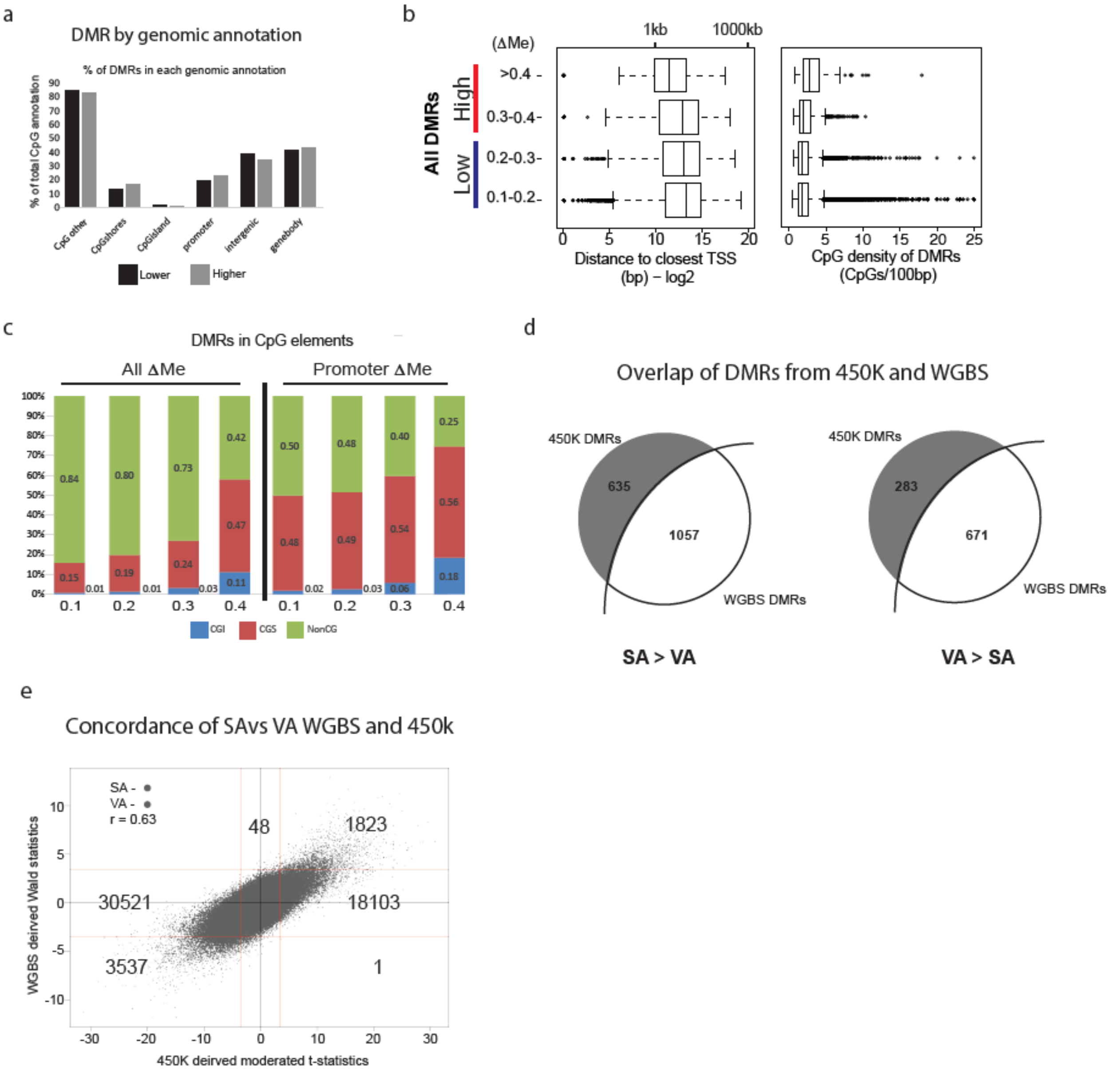

Supplementary Figure S3. DMR characteristics

(a) Distribution of VA- and SA-DMRs with respect to genomic annotation. Y-axis = percentage of total for CpG annotation and genomic annotation separately. **(b)** Box plots stratified by level of ΔMe of DMRs for distance to TSS (left) and CpG density (right). **(c)** Percentage of DMRs by CpGIsland (CGI, blue), CpGShore (CGS, red) or neither CGI nor CGS (NonCG, green) stratified by ΔMe. Left panel for all DMRs and right panel for those within 2kb of TSS. χ^2 calculated for trend across ΔMe groups 0.1-0.2, 0.2-0.3, 0.3-0.4 and >0.4 **(d)** Overlap of 450K and WGBS DMRs. **(e)** Concordance of CpG site methylation t-statistic values between WBGS and 450K SAVA differentially methylated CpG sites. Only sites present on 450k array included.

Association of DMRs with body fat distribution and obesity-related SNPs

IntervalStats (Chikina and Troyanskaya 2012) was used to determine the probability of overlaps or proximity of SA/VA DMRs (67,048) with two sets of SNPs, 97 obesity-related SNPs (Locke et al. 2015) and 50 body fat distribution SNPs (Shungin et al. 2015). The table below summarises the results for the four pairwise combinations of SNPs with DMRs; for each pair there are two

comparisons with either the SNP set or DMR set as the reference and the other the query. The first four columns specify the form of analysis. The last two columns show the FDR corrected minimum (Q) over query intervals, considering all DMRs (ΔMe >0.1) or DMRs with ΔMe >0.2.

Comparison Reference	SNP set	minFDR (DMR diff 0.1)	minFDR (DMR diff 0.2)
SNPs	BMI	1.0000000	Inf
DMRs	BMI	Inf	1.0000000
SNPs	Fat Distribution	0.0356350	0.1476679
DMRs	Fat Distribution	0.1360150	0.1775273

The data shows no relationship between BMI risk SNPs and SA/VA DMRs. However, a suggestive association is seen between SA/VA DMRs and Body fat Distribution. When the analysis is broken down to the highest ranked SNP/DMR pairs for each query direction (Table below), two DMRs are identified that contain body fat distribution SNPs (that the SNPs lie within the DMR is indicated by the negative distance values).

**Associated genes**	**Query Interval**	**Closest Reference Interval**	**Query Lengthbp**	**Distance**	**PValue**	**Q**
**Query by DMR**						
LEKR1	chr3:157079545:157080008	chr3:157079820	464	-188	2.22E-06	0.136015
MEIS1, SPRED2	chr2:65972819:65975685	chr2:65973514	2867	-695	1.82E-05	1
VEG1A	chr11:64095896:64097101	chr11:64095140	1206	756	2.01E-05	1
CMIP	chr16:81499895:81500655	chr16:81501185:	761	530	2.02E-05	1
SFXN2	chr10:102730740:102731104	chr10:102727686	365	3054	4.78E-05	1
**Query by SNP**						
MEIS1, SPRED2	chr2:65973514	chr2:65972819:65975685	1	-695	0.00072725	0.035635
LEKR1	chr3:157079820	chr3:157079545:157080008	1	-188	0.0060174	0.2888352
MACROD1	chr6:6738519	chr6:6736802:6738485	1	34	0.0161739	0.7601733
VEG1A	chr11:64095140	chr11:64095896:64097101	1	756	0.0344767	1
CMIP	chr16:81501185	chr16:81499895:81500655	1	530	0.0501392	1

Ontology of genes associated with DMRs

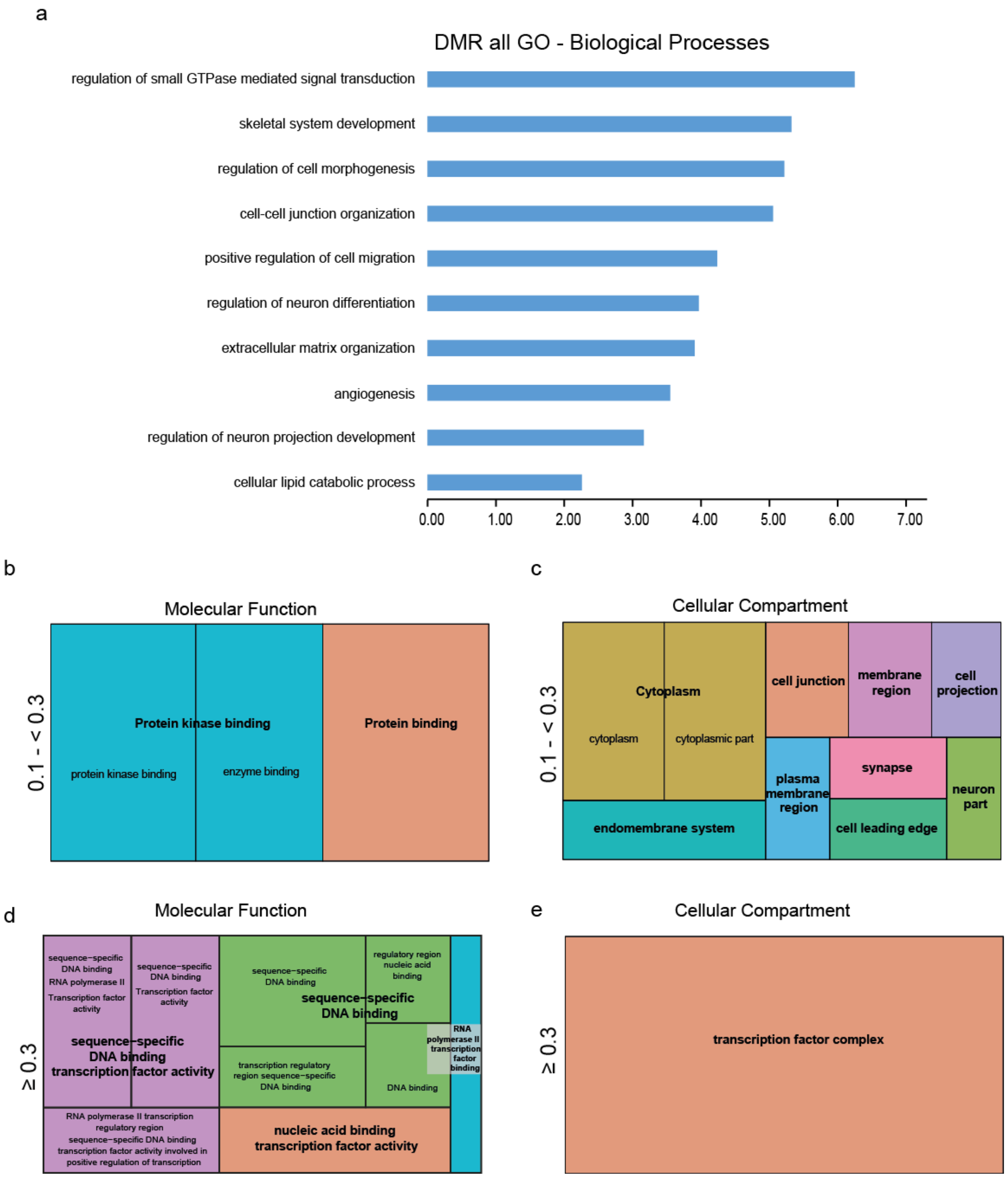

Supplementary Figure S4. Gene ontology SA/VA DMRs.

(a) Gene ontology of biological processes of all SA/VA DMRs. X-axis: Negative log 10 FDR enrichment. **(b,c)** Gene ontology of DMRs with ΔMe between 0.1 and less than 0.3 ΔMe **(b)** tree plot of GO Molecular Function terms identified by Revigo (Supek et al. 2011) **(c)** tree plot of GO Cellular Compartment terms identified by Revigo**. (d,e)** Gene ontology of DMRs with ΔMe greater than or equal to 0.3 ΔMe **(d)** Tree plot of GO Molecular Function terms identified by Revigo. **(e)** Tree plot of GO Cellular Compartment terms identified by Revigo.

DMRs associated with genes encoding Transcription Factors

Given the enrichment of terms relating to DNA binding in the GO analysis we further examined the relationship of DMRs with transcription factor genes. There are many instances in which multiple transcription factor genes from the same family have DMRs within their promoter or gene body. These include – *HOXs*, *PITXs*, *CEBP*s, *DMRT*s, *GATA*s, *IRX*s, and *TBX*s. Examples are shown in Supplementary Figs. 5 and 6. Within most of these families both elevated and reduced methylation in SA compared to VA was observed (**Supplementary Table S12**). However, it is interesting to note that there are a number of gene families where most members have reduced methylation in SA. These include: the key regulator of adipocyte differentiation, *PPARG*, and its family members *PPAR*α and *PPAR*δ and binding partner *RXRA*; the *HIF* family, master regulators of cellular response to hypoxia (*HIF1a*, *ARNT*, *EPAS1*, *ARNT2*, *HIF3a*) along with other related genes encoding basic helix-loop-helix genes *ARNTL*, *ARNTL2* and *SIM1*; and the SMAD family, modulators of numerous signalling pathways (*SMAD1,2,3,4,7,9*).

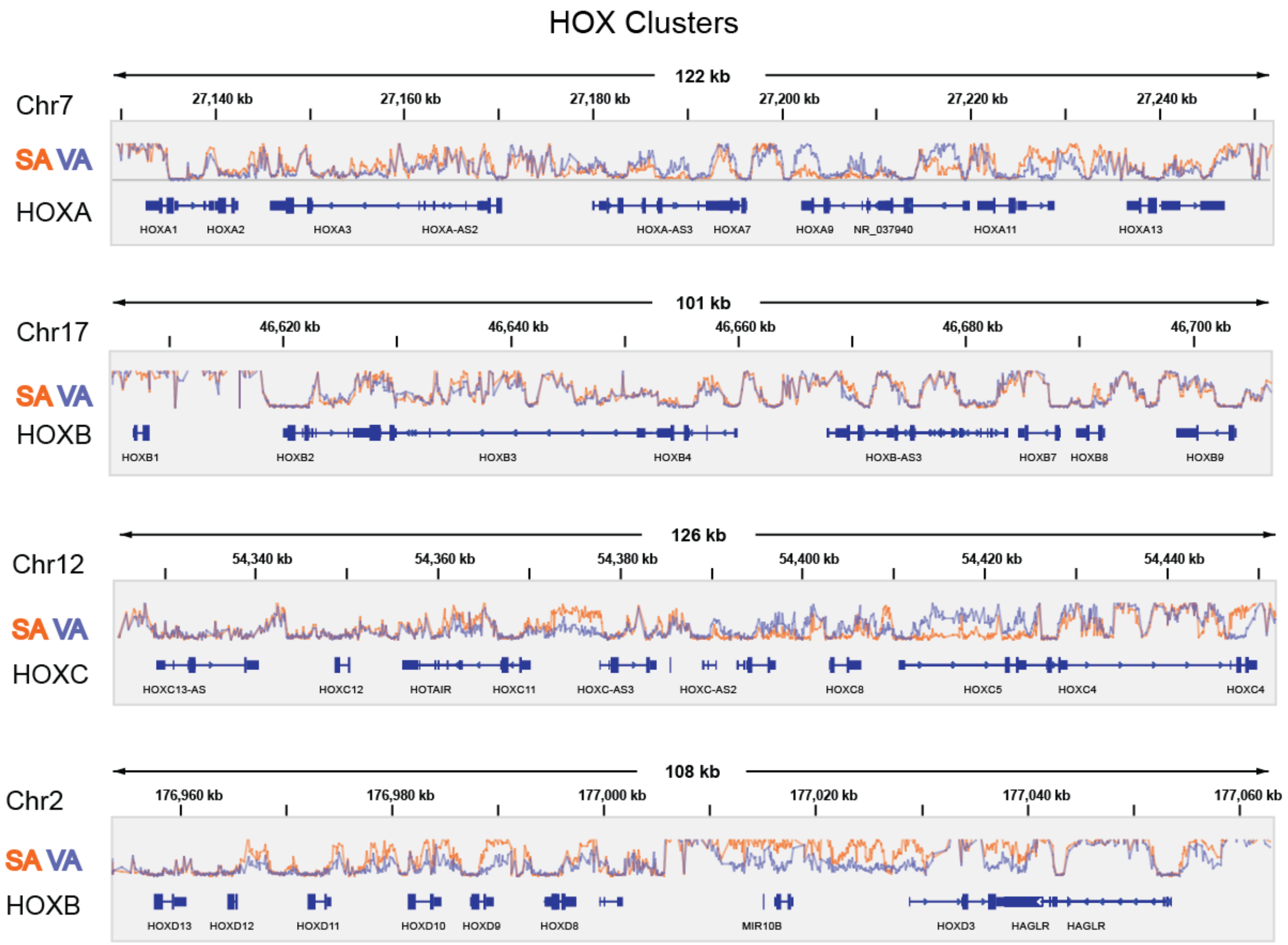

Supplementary Figure S5. Methylation profiles across *HOX* gene clusters. Plots of DNA methylation profiles using the Integrative Genomics Viewer, IGV (Thorvaldsdottir et al. 2013). Complete plot of *HOX* clusters. SA (orange) and VA (purple) DNA methylation levels from 0% to 100%. Chromosomal location top, RefSeq diagrammatic representation of genes bottom tracks.

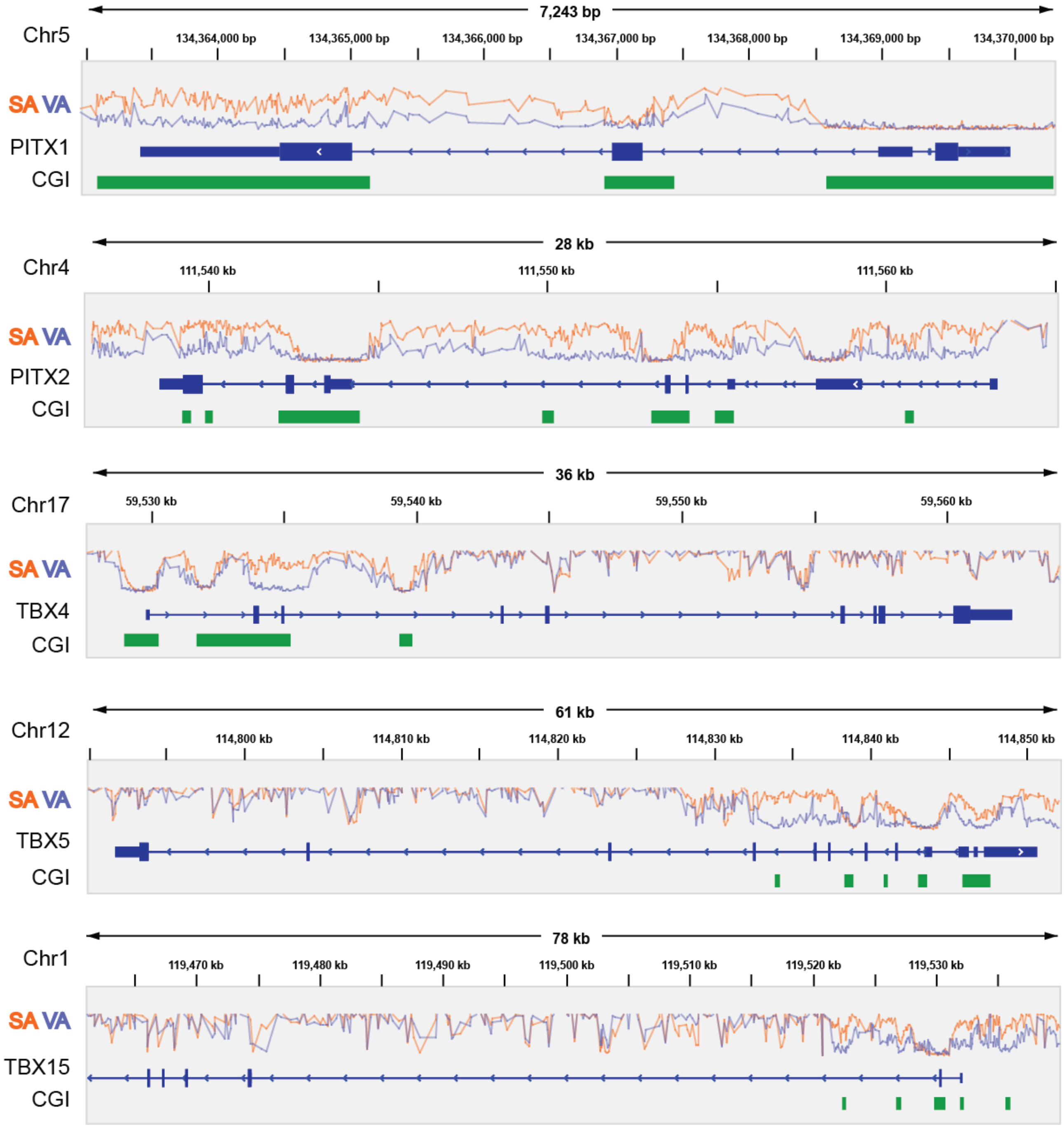

Supplementary Figure S6. Methylation profiles across *PITX* and *TBX* genes.

DMRs associated with *PITX1&2*, *TBX4*,5&15. SA (orange) and VA (purple) DNA methylation levels from 0% to 100%. CGI = CpG Islands (green blocks). For details see Supplementary Figure S5.

Relationship of DNA methylation and DMRs with gene expression

To broadly characterize the relationship between DNA methylation and gene expression in SA and VA we examined the DNA methylation profile around the TSS of all annotated gene promoters. As has been noted for other tissues (Roadmap Epigenomics et al. 2015) decreasing DNA methylation at the TSS was associated with progressively increased gene expression (Supplementary Fig. S7a). This association is particularly evident for promoters with medium and high CpG content.

Notably, lowest methylation levels were observed about 200 bp after the TSS. Also notable for the most highly expressed genes was the increased levels of methylation both upstream and downstream of the TSS (for more detail with respect to CpG density and gene expression see Supplementary Material and Supplementary Fig. S7b). Interestingly, promoters with low CpG density show a more heterogeneous relationship with gene expression and methylation relative to promoters with higher CpG density.

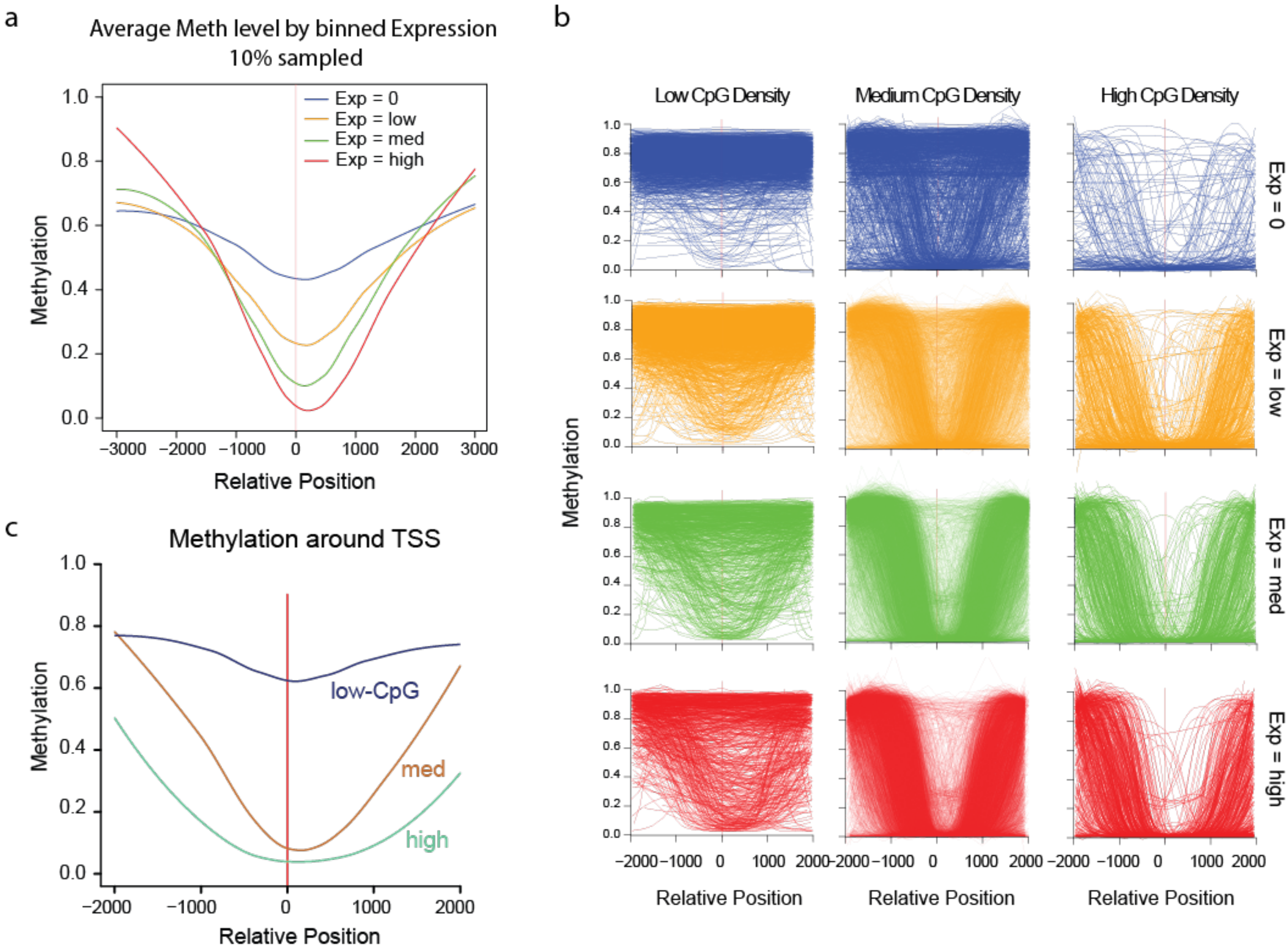

Supplementary Figure S7. Relationship of Promoter DNA methylation with gene expression. (a) Genes were grouped based on VA expression level into non-expressing (blue), and those in the low (yellow), medium (green) and high (red) thirds. Loess regression was used to fit a curve representing overall methylation levels in VA 3kb either side of the TSS of these grouped sets of genes. (**b**) Gene sets were further split by CpG density of promoter region into Low (<70 CpGs across 4kb region), medium (70 to 230 CpGs across 4 kb) and high (>230 CpG across 4 kb) CpG density groups (Weber et al. 2007). Plots show overlayed curves of VA DNA methylation across

individual promoters in each group. Colour-coding by gene expression level as in (a). (**c**) As for Panel (a), but with promoters grouped by CpG density, low (blue), medium (orange) and high (green).

Features and Functions of DE-DM Genes

The visceral fat depot releases more free fatty acid than subcutaneous fat due to lower sensitivity to insulin to suppress lipolysis (Arner 2005). We found that genes associated with lipid droplet and lipid mobilization such as *CIDEC*, monoglyceride lipase (*MGLL*), triglyceride lipase (*PNPLA3*) and *PLIN4* all have higher promoter methylation and reduced expression in VA. Other key genes in metabolic regulation such as *CPT1A* and *PDK4*, exhibit both elevated expression and promoter methylation in VA. Numerous developmental genes associated with fat distribution and waist-hip ratio were DE-DM (Fig. 3d and Supplementary Table S14), these included *HOXA11* and *TBX15* that had both higher expression and DNA methylation in SA(Shungin et al. 2015). Fat distribution is highly regulated by the presence of sex hormones and it has been shown that increased estrogen (17β-estradiol) in abdominal SAT can potentiate lipolysis (Gavin et al. 2013). Interestingly we found that both estrogen receptors 1 and 2 show higher expression and lower methylation in SA. Differences were also observed for mesenteric estrogen-dependent adipose gene, MEDAG, which has been identified as a novel gene of visceral adiposity (Zhang et al. 2012). Angiogenesis and extra cellular matrix (ECM) remodeling are crucial processes for proper formation of adipose tissue, and vascularisation and angiogenic capacity are known to differ between different fat depots (Gealekman et al. 2011). A number of DE-DM genes in our data set are involved in angiogenesis, hypoxia and ECM remodeling (Fig. 3d and Supplementary Table S14). One of these genes is hypoxia inducible factor HIF3A, for which elevated methylation in blood and subcutaneous fat was found to be associated with obesity/BMI (Dick et al. 2014).

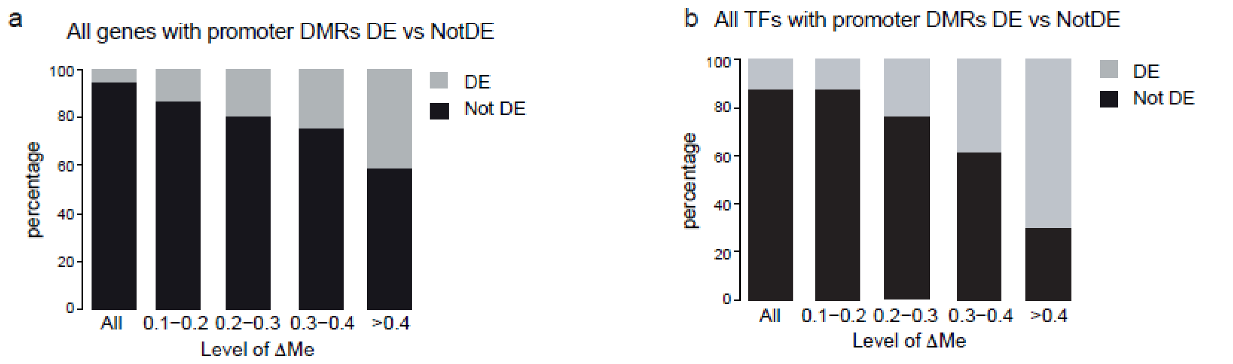

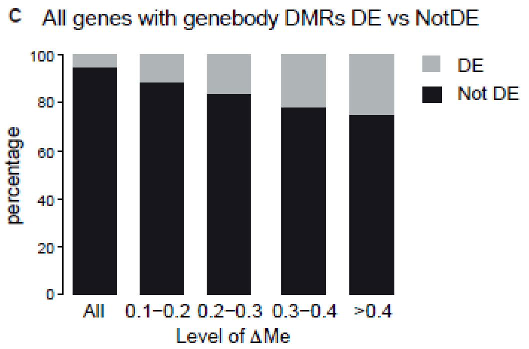

d

Gene Expression and DNA mehtylation	Number of genes	Number of TF genes (%)	Number of gene promoters overlapping DMVs (%)	CpG density of promoter	CpG dentisty of DMR
**Concordant**	173	31 (18.0)	27 (15.6)	4.8	2.9
**Opposite direction**	287	31 (10.8)	33 (11.5)	5.0	2.5
Significance (Fisher Exact test)		0.035	0.253	N/A	N/A

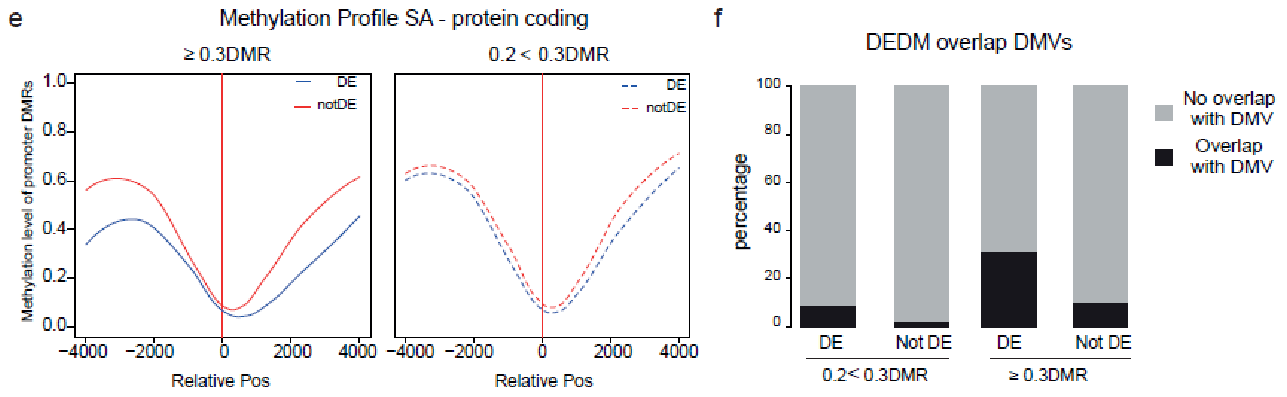

Supplementary Figure S8. Features of DE-DM genes.

(a) Barplots of Refseq genes split by whether they are differentially expressed (grey) or not (black). Plots are shown for all genes (left) and separated into those with promoter DMRs between 0.1 and <0.2, 0.2 and <0.3, 0.3 and <0.4, >0.4. **(b)** As for Panel (a), but for genes encoding Transcription Factors **(c)** As for Panel (a), but for gene-body DMRs. **(d)** Association of different features with concordant compared with discordant promoter methylation and gene expression **(e)** Plot of methylation level in SA for promoter DMRs. Plots are stratified into 4 groups: differentially expressed (DE) and Low DMRs (ΔMe = 0.2 to <0.3 – blue dashed line); DE and High DMRs (ΔMe ≥ 0.3 – blue solid line); Not DE and Low DMRs (ΔMe 0.2 to <0.3 – red dashed line); Not DE and High DMRs (ΔMe ≥0.3 – red solid line). Loess regression was used to fit a curve representing overall methylation levels in SA, 4kb either side of the TSS, of these grouped genes. X-axis = relative position to the TSS of genes, y-axis = methylation level. **(left)** High DMRs - ΔMe ≥0.3, **(right)** Low DMRs - ΔMe 0.2 to <0.3**.** **(f)** Barplots of overlaps of DMRs with DMVs. Percentage with overlap of DMR and DMV shown in black, and without overlap in grey. DMRs are segregated into those with Low (0.2-<0.3) or High (≥0.3) ΔMe and whether the associated gene is DE or not.

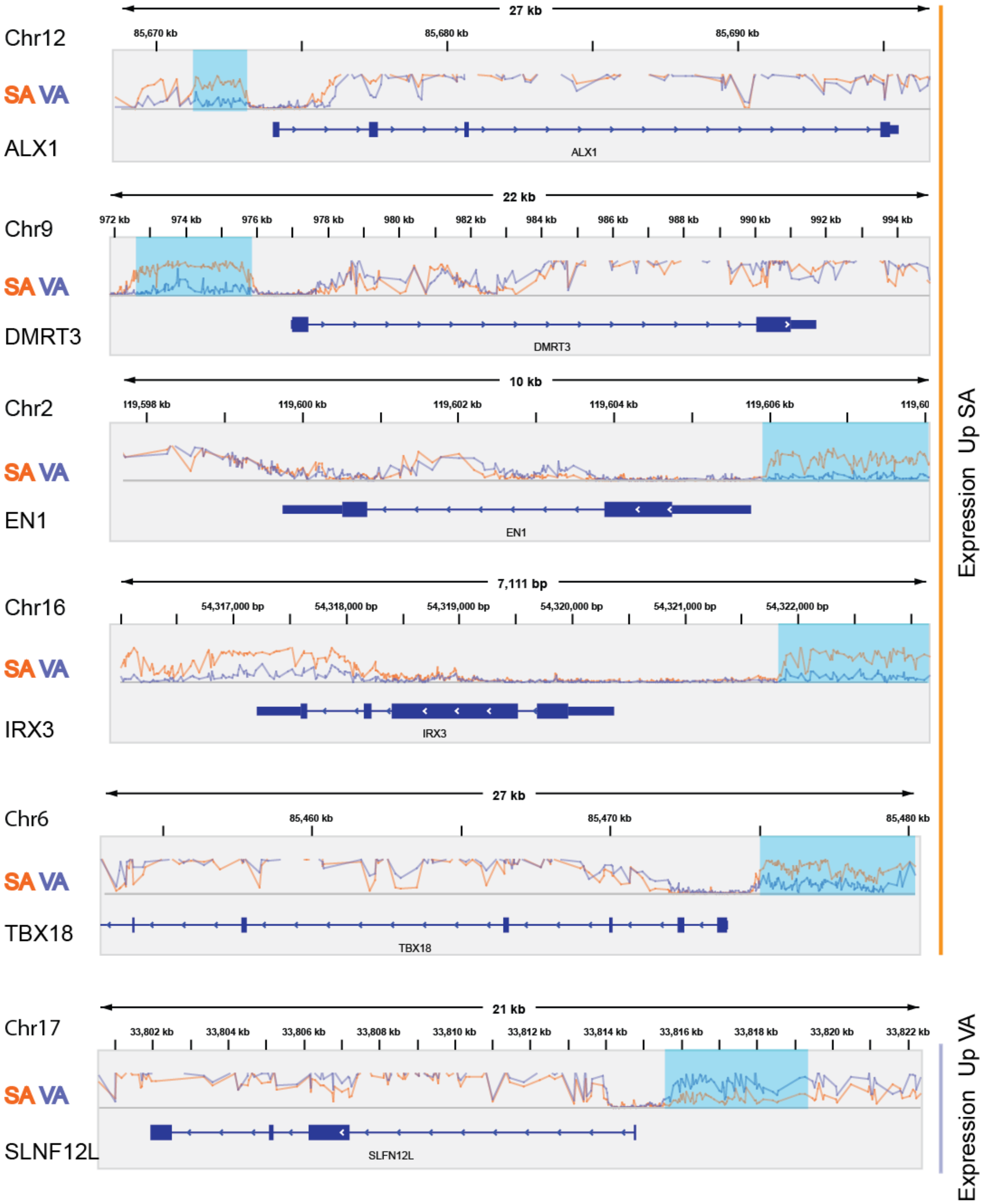

Supplementary Figure S9. Examples of Methylation Cliffs. Plots of DNA methylation profiles using the Integrative Genomics Viewer (14). DNA methylation cliffs (outlined in blue) associated with *ALX1*, *DMRT3*, *EN1*, *IRX3*, *TBX18* and *SLFN12L*. SA (orange) and VA (purple) DNA methylation levels from 0% to 100%. Chromosomal location top, RefSeq diagrammatic representation of genes bottom tracks. Side bars denote direction of differential expression, orange is higher in SA and purple is higher in VA.

Regulatory elements identified within DNA methylomes of purified SA and VA

Putative regulatory elements (UMRs and LMRs) as well as DMVs were identified from WGBS data separately for each sample (for each cell type from each individual). The numbers of regions identified are shown in **Supplementary Fig. S10a**. Venn diagrams showing the overlaps of scored UMRs and LMRs between the three individuals are shown in **Supplementary Fig. S10b**. All individual samples had approximately 20,000 UMRs with a strong overall concordance within each sample type (>87% in common). Between-sample calling of LMRs was more variable than UMRs with about 50% being called in a given sample type for all three individuals and a further ~18-22% being called in 2 of 3 individuals. For comparisons between cell types, regions being scored in at least two individuals are included in **Supplementary Tables S15-S20**.

The averaged DNA methylation profiles across UMRs, LMRs and DMVs for each of SA, VA and PBL are very similar (**Supplementary Fig. S10c**).

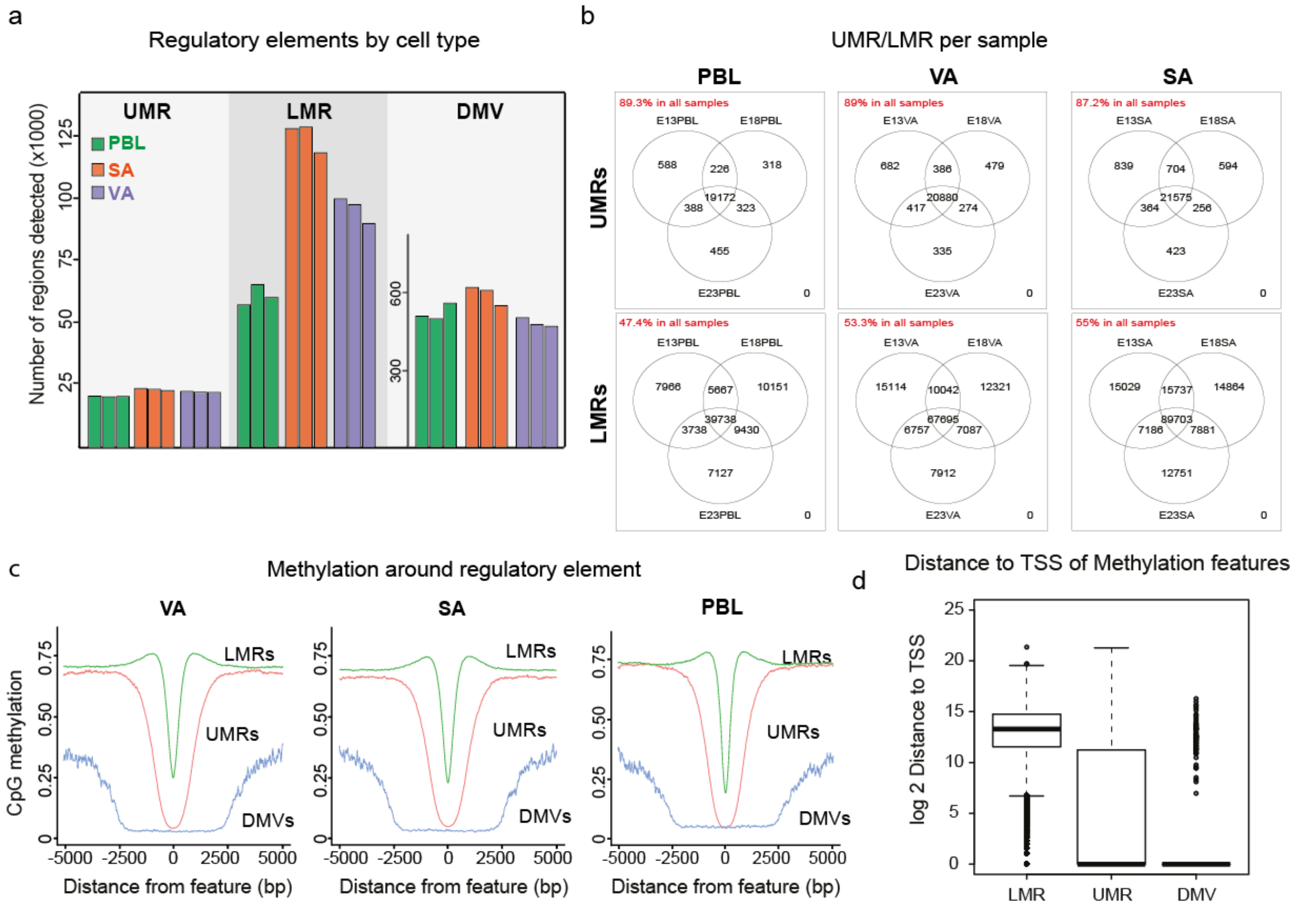

Supplementary Figure S10. UMR, LMR, DMV analysis.

(a) Number of UMRs, LMRs and DMVs in SA, VA and PBL for three subjects. **(b)** Venn diagrams of the numbers of UMRs, LMRs and DMVs identified in each sample, and in common between samples. **(c)** Smoothed averages of methylation levels of LMRs (green), UMRs (red) and DMVs (blue) centred around midpoints of each feature for VA, SA, PBL. **(d)** Box plots of distances of UMR, LMR and DMV features to nearest TSSs.

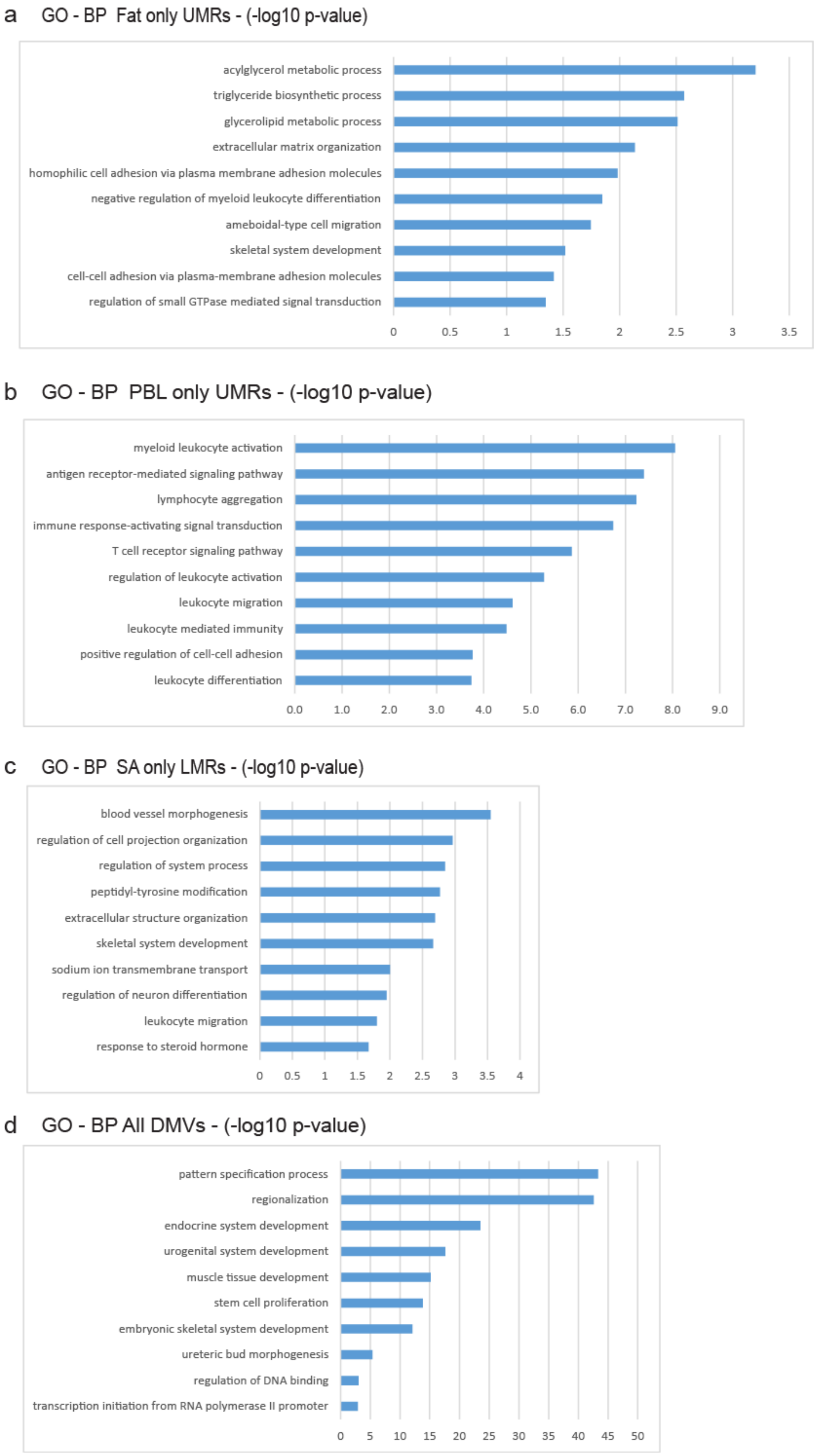

Supplementary Figure S11. Selected Gene Ontologies for UMRs, LMRs and DMVs.

(a) Gene ontology of UMRs identified in fat samples (i.e. UMRs identified in both SA and VA, but not PBL). **(b)** Gene ontology of UMRs identified in PBL samples only. **(c)** Gene ontology of LMRs unique to SA. **(d)** Gene ontology of all DMVs, enrichment values for selected terms in negative log 10. For all panels, X-axis is negative log 10 of FDR value for over representation.

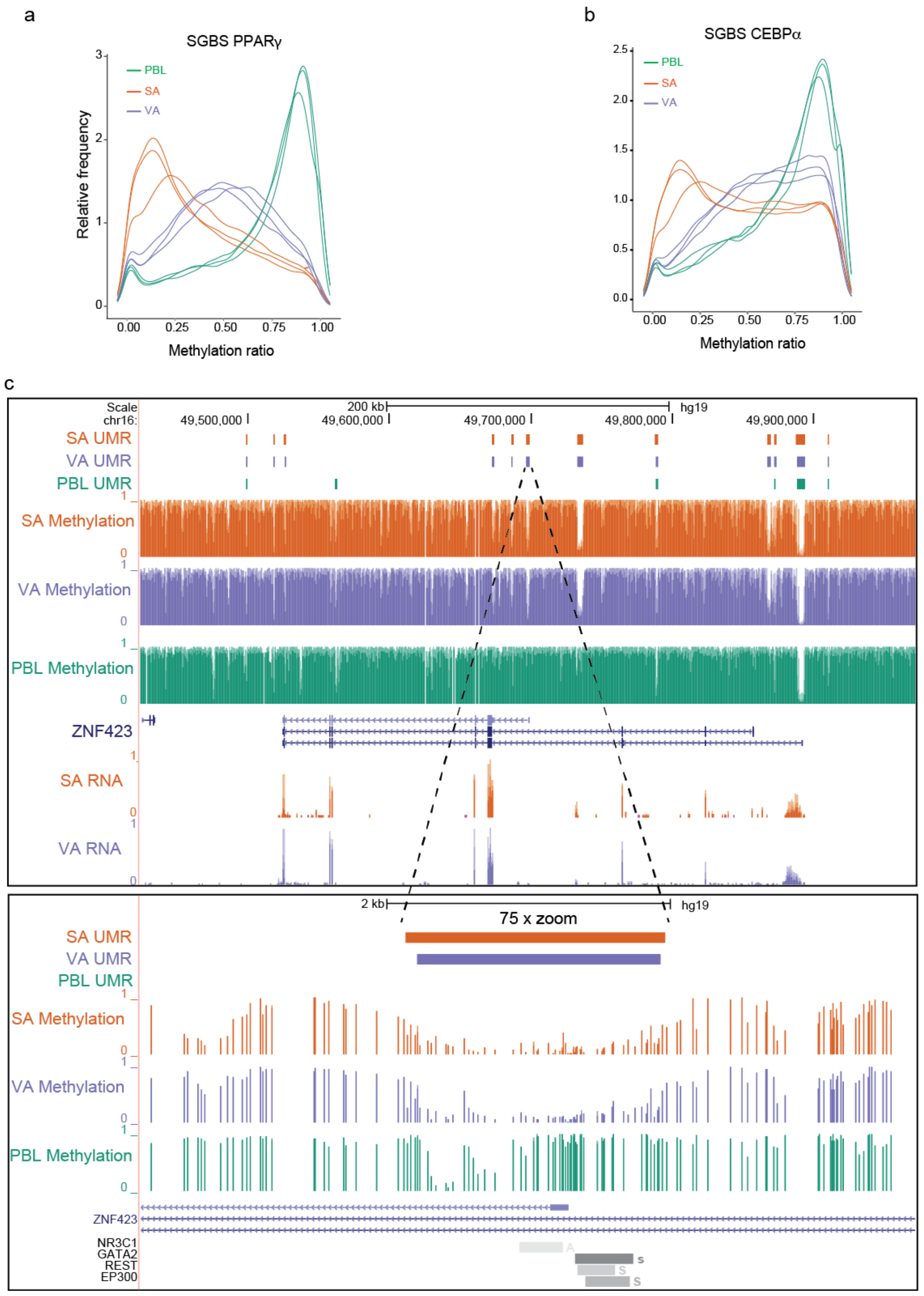

Supplementary Figure S12. Regulatory elements and TF binding.

(**a**) Density plot of SGBS PPARγ ChIP-seq peaks (16) against the level DNA methylation within the regions determined from our SA, VA, PBL WGBS data. **(b)** Density plot of enrichment for SGBS CEBPα ChIP-seq peaks (Schmidt et al. 2011) by DNA methylation ratio in our SA, VA, PBL WGBS data. **(c)** UCSC browser image of *ZNF423*. Upper panel = broad view (1x zoom), Lower panel = zoomed in view (75x zoom): UMRs, WGBS and RNA-seq profiles shown as orange, purple and green respectively for SA, VA and PBL. Transcription factor ChIP-seq peaks shown as grey bars.

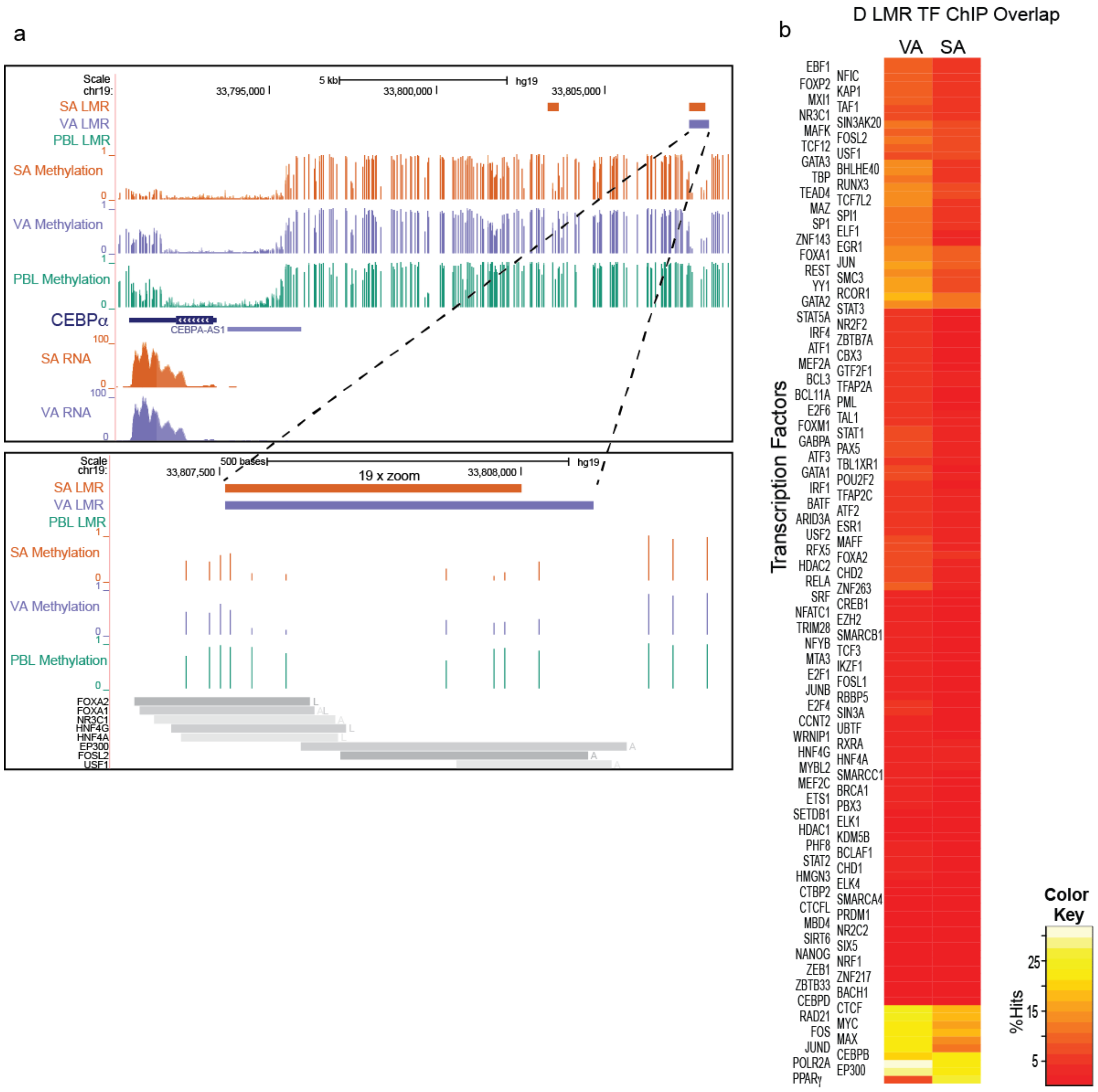

Supplementary Figure S13. LMRs and TF binding.

(a) UCSC browser image of *CEBPα*. Upper panel = broad view, Lower panel = zoomed in view: LMRs, WGBS and RNA-seq profiles shown as orange, purple and green respectively for SA, VA and PBL. Transcription factor ChIP-seq peaks shown as grey bars. **(b)** Heatmap comparing the percentage of SA D-LMRs (differential LMRs, right column) that contain a ChIP-seq peak for a particular TF, with those of VA D-LMRs (left column). Red = low percentage, and white = high percentage, of TF overlap.

Clustering of TF binding sites in D-LMRs

To identify potential regulatory regions distinguishing SA and VA, we chose LMRs that were in the lowest 25% of methylation and specific to either SA or VA. We further overlapped these with VA-SA DMRs. ENCODE ChIP-seq binding sites (162 TFs) and subcutaneous adipocyte PPARγ ChIP-seq binding sites were then mapped onto these DMRs. Binding sites for individual TFs within SA and VA-specific DMR/LMRs are shown in **Supplementary Tables S26 and S27**, and unsupervised clustering of TF binding and individual regions in **Figure 5c and Supplementary Fig. S14a**. Clusters of regions containing binding sites for common set of TFs are indicated in the figures. Clusters containing similar sets of TFs in SA and VA are indicated by letters A-F.

a

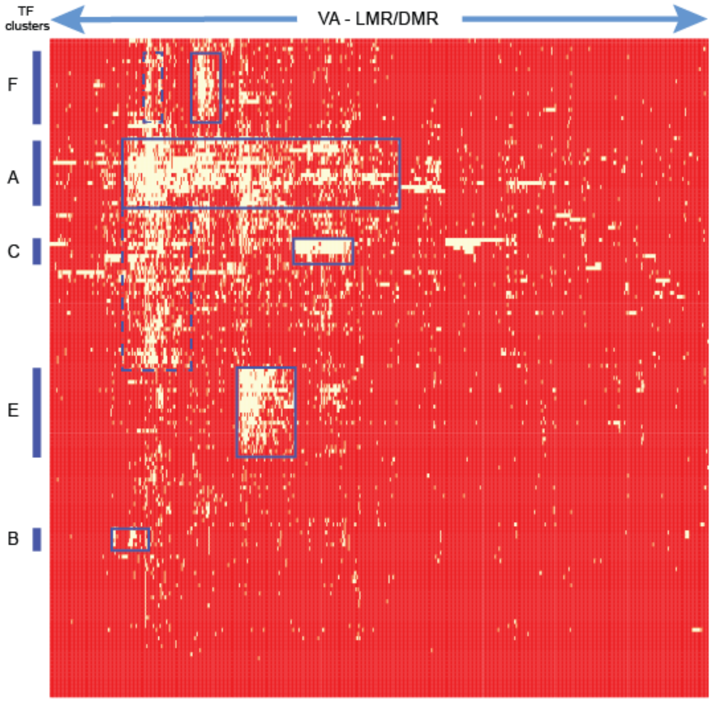

b

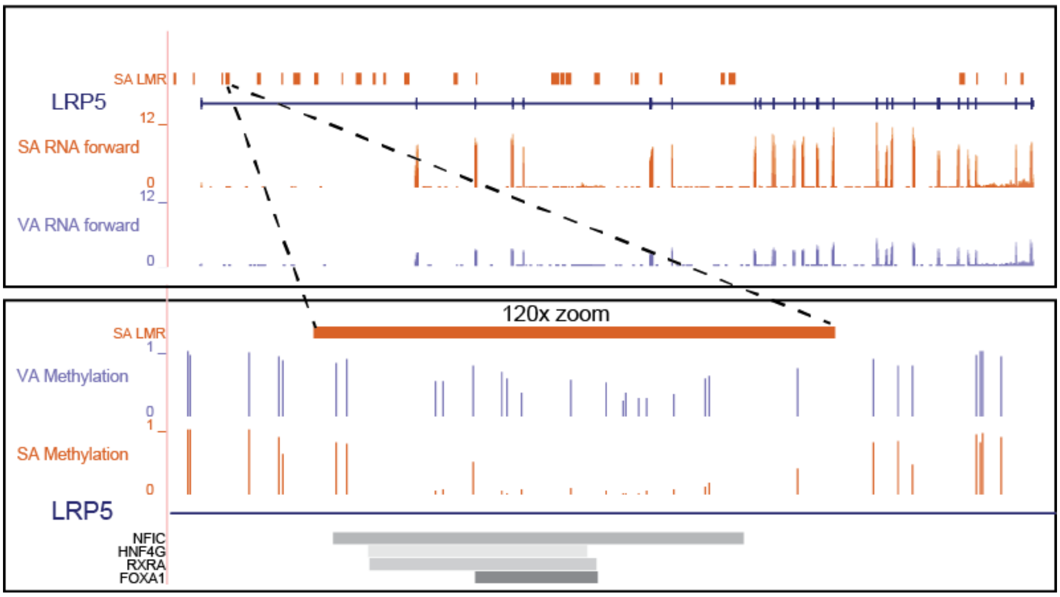

Supplementary Figure S14. TF binding to D-LMRs.

(a) Unsupervised cluster plot of the presence (yellow) or absence (red) of ChIP-seq peak for 162 TFs (vertical axis) within 862 VA D-LMRs (horizontal axis). TF clusters A-F represent clusters of regions containing binding sites for common set of TFs. See table immediately following for detail. **(b)** UCSC browser image of *LRP5*. LMRs, WGBS and RNA-seq profiles shown as orange, purple and green respectively for SA, VA and PBL. Transcription factor ChIP-seq peaks shown as grey bars.

The TFs in each cluster are shown in the table below, with those in common between SA and VA shown in bold. TFs contributing to these clusters are discussed further below.

Cluster	SA LMRs/DMRs	VA LMRs/DMRs
A	**CEBPB, STAT3**, **EP300, POL2RA**, **MAX, MYC**, **FOS, JUN, JUND**, FOSL2, PPARG	**CEBPB, STAT3**, **EP300, POL2RA**, **MAX, MYC**, **FOS, JUN, JUND**, BHLHE40, RCOR1, MAZ, YY1, TEAD4
**B**	**CEBPD, HNF4A, HNF4G, MYBL2, RXRA,** ARID3A, FOXA1/FOXA2, HDAC2, TEAD4	**CEBPD, HNF4A, HNF4G, MYBL2, RXRA,** MBD4
C	**CTCF, RAD21, SMC3, ZNF143**	**CTCF, RAD21, SMC3, ZNF143**
D	BRCA1, GTF2F1, RFX5, STAT1, TCF7L2, TFAP2A, TFAP2C, SMARCC1, SMARCC2	
E	**BCL11A, IRF4, POU2F2, BATF, RELA, PAX5, RUNX3, TCF3**	**BCL11A, IRF4, POU2F2, BATF, RELA, PAX5, RUNX3, TCF3,** NFIC, MEF2A, MEF2C
E-extended	**MEF2A, MEF2C**, **MTA3, NFATC1, IKZF1**, **EBF1,** PML, STAT5A, TBL1XR1, SPI1	**MTA3, NFATC1**, **IKZF1, EBF1,** PBX3, BCLAF1, BCL3, WRNIP3
F	**CBX3**,**TRIM28, NR2F2**, **TAL1,** ATF1, FOSL2, JUNB, IRF1, GATA1	**CBX3**,**TRIM28, NR2F2**, **TAL1,** GABPA, ETS1, ZBTB7A, CCNT2, HMGN3, PML, STAT5A, TBL1RX1

Cluster A

A large number of regions bind the one or both of the key adipocyte TFs, PPARG and CEBPB as well as a set of set of common transcriptional regulators, EP300, POL2RA, MAX, MYC, FOS/JUN family members and STAT3. In different regions these core components are often found in association with other TFs, eg., NR3C1(Glucocorticoid receptor), USF1 and 2, GATA2 & 3 and FOXP2 (extended dotted region) or with other clusters (eg. Clusters D and B)

Cluster B

For both SA and VA this cluster includes MYBL2, HNF4A, HNF4G and RXRA (the heterodimer binding partner of PPARγ) and binding is also commonly associated with PPARγ and/or CEBPδ. In SA other TFs in this cluster are NFIC, SP1, CEBPD and TEAD4. In post-natal life, members of the FOXA family control glucose metabolism through the regulation of multiple target genes in the liver, pancreas, and adipose tissue. FOXA proteins function as 'pioneer factors' whose binding to

promoters and enhancers enable chromatin access for other tissue-specific transcription factors (Friedman and Kaestner 2006). NFIC has recently been shown to regulate adipocyte differentiation through the canonical wnt pathway (Zhou et al. 2017) and to bind to and activate the *LRP5* promoter in mice. Among SA LMR/DMRs, we identified a candidate regulatory region in the first intron of the *LRP5* gene (about 1.2 kb downstream of the TSS) that contains ChIP binding sites for NFIC, RXRA, HNF4G and FOXA1 and that could contribute to the higher expression of LRP5 in SA compared with VA (**Supplementary Fig. S14b**).

Cluster C

This includes the chromatin structural proteins CTCF, RAD21 and SMC3 along with ZNF143 and YY1, indicative of regions that are involved in connecting promoters with distal regulatory elements. ZNF143 provides sequence specificity to secure chromatin interactions at gene promoters (Bailey et al. 2015). CTCF, RAD21 and SMC3 are components of the cohesion complex and orchestrate the mitotic chromatin interaction landscape (Kakui and Uhlmann 2017).

Cluster D

While segregating as a separate cluster in SA, the TFs in this cluster are commonly associated in SA LMR/DMRs with Cluster A TFs. TCF7L2 is a major wnt pathway TF and repressor of adipogenesis, while AP2 (TFAP2A and TFAP2C) represses adiponectin expression and has roles in glucose uptake. In VA many of these TFs also form part of an extension of Cluster A; for example TCF7L2 binding sites are found in 114 VA LMR/DMRs of which 75 also contain EP300 ChIP sites (**Supplementary Table S27**).

Cluster E

Cluster E also contains TFs important in adipocyte function. IRF4 is important in control of lipid handling (Eguchi et al. 2011) while ATF2 is critically involved in early steps of adipogenesis (Maekawa et al. 2010). EBF1 regulates adipocyte morphology, lipolysis and inflammatory pathways (Griffin et al. 2013; Gao et al. 2014). STAT5A also has a significant function in promoting adipogenesis and regulates expression of a number of genes, including adiponectin (Able et al. 2017).

Cluster F

This cluster contains four genes in common between SA and VA LMR/DMRs, CBX3, TRIM28, NR2F2 and TAL1. TRIM28 is an epigenetic regulator for which haploinsufficiency has been implicated in obesity (Dalgaard et al. 2016) and that is also associated with CBX3 as a key regulator of nephrogenesis in mice (Dihazi et al. 2015). NR2F2 (COUPTFII) has been previously described as an essential regulator of adipogenesis (Xu et al. 2008). In addition of Cluster F TFs, D-SA LMRs that contain NR2F2 sites show high frequency of binding sites for a number of sequence-specific TFs including TEAD4, GATA2, STAT5A as well as CEBPβ and PPARγ.

Materials and Methods

CONTACT FOR REAGENT AND RESOURCE SHARING

Further information and requests for resources and reagents should be directed to and will be fulfilled by the Lead Contact, Susan Clark (s.clark@garvan.org.au)

SUBJECT DETAILS

Subjects were recruited from April 2012 to November 2013. All subjects underwent elective Laparoscopic Cholecysectomy surgery at St Vincent's Hospital, Sydney. The study protocol was approved by the St Vincent's Hospital Human Research and Ethics Committee, SVH File Numbers: H06/151 and 12/200. All subjects gave informed written consent to participate. The following selection criteria were used to identify participants in the study: Age – men or pre-menopausal woman aged 35–50 years; Weight: 18 – 26.0 kg/m^2 (females), 23.3/27.9 kg/m^2 (males); FBG: < 5.6 mmol/l; no history of malignancy or other severe medical illness: Willingness to give written informed consent and willingness to participate in and comply with the study. The physical and metabolic measures of the subjects are summarized in **Supplementary Table S1**.

METHOD DETAILS

Blood collection

Venous blood was collected on the day of elective surgery from the antecubital vein following an overnight fast from midnight. Samples were collected for glucose, lipids, CRP and circulating leukocytes were isolated for DNA methylation measurements. For the latter, 10 mL blood was collected into K2EDTA vacutainers. Following centrifugation at 1,300 rcf for 10 minutes at room temperature, the top plasma layer was removed and the buffy coat layer collected. Cells were washed three times, with vigorous resuspension, in 10 mL Tris-EDTA buffer (100 mM Tris-HCl, 0.1 mM EDTA), with pellets collected after centrifugation at 10,000 rcf for 10 minutes. The final pellet was resuspended in 500 µL of buffer and stored at -80ºC. Blood biochemistry was performed by Sydpath – the Pathology Service of St Vincent's Hospital Sydney, Australia.

Adipose tissue collection

Visceral adipose tissue (VAT) was collected from the greater omental region; subcutaneous adipose tissue (SAT) from the periumbilical site of surgical incision. Adipose samples were separated into two samples: one was snap frozen for whole tissue analysis; the second was prepared for adipocyte isolation – see below.

Adipocyte isolation

Adipocyte isolation was adapted from (Rodbell 1964; Reynisdottir et al. 1994). In brief, freshly isolated VAT and SAT samples were weighed and minced with surgical scissors. For each gram of minced tissue, 1.25 mg/mL of Collagenase I (Sigma Aldrich, CAT#C6885-1G) was added to 2 mL of HEPES buffer containing 7.9 g/L NaCl, 323 mg/L $CaCl_2.2H_2O$, 308 mg/L $MgSO_4.7H_2O$, 154.3 mg/L Na_2HPO_4 (anhydrous), 5 mL/L of 1M Hepes (Sigma Aldrich), 450 mg/L D-glucose, pH 7.2 – 7.4, 20 g/L BSA. The tissue was incubated for 30-45 minutes at 37ºC before being diluted 1:9 with additional HEPES buffer. The mixture was passed through a 250 micron mesh before being

centrifuged at 300 rcf for 6 minutes at room temperature. The top lipid layer was removed and the adipocytes collected for snap-freezing in liquid nitrogen.

RNA isolation

RNA was extracted from VA and SA sample types following the RNeasy Lipid Tissue Mini Kit (Qiagen, Cat#74804), as we have described before. The protocol was followed exactly except that homogenisation used the probe tissue ruptor, and on-column DNase digestion was carried out. RNA quality and quantity was determined on the Agilent Bioanalyser.

DNA isolation

DNA from the VA and SA sample types was extracted using an in-house method. For every 200 – 350 µL of frozen sample, 1 mL of TP lysis buffer (Tris 50mM pH 7.5, NaCl 0.1M, SDS 0.5%, EDTA 5mM) and 100 µL of proteinase K (20 mg/ mL, Promega, Cat#V3021) was applied. This solution was then incubated overnight at 55ºC with constant shaking (700 rpm). The next day, after centrifugation at 2,000 rpm, the lipid layer was discarded and an equal volume of phenol: chloroform: isoamyl alcohol was applied. After centrifugation the aqueous phase was collected and precipitated in 0.3M sodium acetate, 66% v/v ethanol and 100 mg/ mL Glycoblue (Life Tech, cat#AM9515) while incubating in dry ice for 30 minutes. The DNA was pelleted and washed in 75% ethanol before being resuspended in 100 µL milliQ water.

VAT DNA was extracted using the DNeasy Blood and Tissue Kit (Qiagen, Cat#69504) following an amended protocol for total DNA from animal tissue. Briefly, 30 mg pieces of tissue were homogenised in 360 µL of ALT buffer using the bead beater for 2 minutes set at full speed, and a 5 mm stainless steel bead (Qiagen, Cat#69989). 40 µL of Proteinase K was added and the solution incubated at 56ºC overnight with constant shaking (500 – 700 rpm). 400 µL of Buffer AL was mixed with the sample the following day and this mixture was passed through the DNeasy Mini spin column. The adherent DNA was then washed with Buffers AW1 and AW2 and then eluted in 200 µL of Buffer AE.

DNA was extracted from PBL sample types following the Gentra Puregene Blood Cell Kit (Qiagen, Cat#158445).

Whole Genome Bisulfite Sequencing (WGBS)

Whole genome bisulfite sequencing libraries were prepared following Illumina's "Whole- Genome Bisulfite sequencing for Methylation Analysis" protocol. Briefly, 1 µg of genomic DNA was spiked with 0.5% unmethylated lambda DNA and sonicated to generate fragments of size between 150 to 300bp. Library preparation was performed for using the Illumina's Paired-end DNA Sample Prep Kit (discontinued, Illumina, CA, USA) according to the manufacturer's protocol. The size selected libraries were then subjected to bisulfite conversion as previously described (Clark et al. 2006). Adaptor-ligated bisulfite treated DNA was enriched by 10 cycles of PCR amplification using the PfuTurbo Cx Hotstart DNA Polymerase (Stratagene). Qualitative and quantitative checks of the libraries were performed using Agilent's High sensitivity DNA kit (Agilent) and KAPA Library quantification kit (KAPA Biosystems). Three lanes of paired end 100bp sequencing was performed for each of the library on the Illumina HiSeq2500 platform using the TruSeq v3 cluster kits and SBS kits to achieve coverage ranging between 25-30x.

Gene Expression

Poly-A RNA-seq

RNA-seq libraries were prepared using Illumina TruSeq Stranded poly-A RNA Library Prep Kit by the University of Western Sydney Next Generation Sequencing Facility. 500 ng of RNA was used for each sample. Libraries were sequenced using 100bp paired-end HiSeq2500 chemistry3 by UWS, yielding at least 30 million reads per sample, **Supplementary Table S1**.

Whole RNA-seq

RNA-seq libraries were prepared from 500 ng RNA, following ribosomal RNA reduction using Illumina TruSeq Stranded Total RNA Library Prep Kit by the Australian Genome Research Facility (AGRF). Libraries were sequenced using 100bp paired-end HiSeq2500 chemistry4 by AGRF, yielding 35 to 40 million aligned reads per sample, **Supplementary Table S1**.

QUANTIFICATION AND STATISTICAL ANALYSIS

Illumina 450K analysis

Briefly normalization was performed using the *dasen* method from watermelon (Pidsley et al. 2013). Some probes were excluded from the analysis. In particular these were, the union of probes on the sex chromosomes (n = 11,648), probes targeting CpGs located two or fewer nucleotides from a known single nucleotide polymorphism (SNP) with a minor allele frequency >0.05 (n = 29,476), and known cross-hybridizing probes (n = 30,969) published by Chen et al (Chen et al. 2013). Probes were also excluded if they failed quality control metrics in one or more samples, based on a detection P value >0.05.Probe M-values were used to detect differentially methylated probes via Limma (Ritchie et al. 2015). Differentially methylated region calling was performed with DMRcate (Peters et al. 2015), using default parameters.

WGBS

Alignment (Normal weight)

Reads were aligned using https://github.com/clark- lab/Bisulfite_tools. Briefly, paired- end reads were adaptor and quality score trimmed using trim_galore v0.2.8. Trimmed reads were mapped to the hg19 genome build using bismark v0.8.3 (Krueger and Andrews 2011) with the parameters "- - bowtie2 - X 1000", technical replicates pooled using samtools v0.1.19 "merge" and PCR duplicates marked using Picard v1.91 "MarkDuplicate s". Methylation count data is extracted using bismark_methylation_extractor with the parameters "- - comprehensive - - merge_non_CpG - - bedgraph - - counts - - report - - gzip - - buffer_size 20G". Methylation data is joined for each sample into a ~28M row "bigTable" containing methylated and total read counts for each CpG site in the genome.

Calling methylation regions within each sample

UMRs and LMRs were called using the MethylSeekR Bioconductor package (Burger et al. 2013) with the parameters "meth.cutoff=0.5, nCpG.cutoff=5" with no PMD filtration. DMVs were called using the approach described in (Hovestadt et al. 2014), implemented in the R package 'aaRon' (https://github.com/astatham/aaRon) 'findDMVs' function using default parameters.

Calling methylation changes between groups of samples

DMRs were called using the bsseq Bioconductor package (Hansen et al. 2012). Smoothing was performed using the parameters "ns=70, h=1000" and only CpG sites with minimum 2x coverage in at least 2 samples in each group being compared was used. T- stats were called using the parameters "estimate.var='paired', local.correct=TRUE" and the DMRs called using a t- stat cutoff of 4 and filtered for minimum 3 CpG sites and an absolute change in average methylation of 10%. Given our sequencing depth across 3 biological replicates 10% DMRs should have a true positive rate (TPR) of >80% and false discovery rate (FDR) < 10%, while 20% DMRs TPR >90% and FRD <5% (Ziller et al. 2015).

Identifying and plotting enrichment for TF binding

The significance of ChIP-seq peak overrepresentation at DMRs/LMRs/UMRs was calculated using the hypergeometric test (phyper) in R. Publically available ENCODE ChIP-seq peaks were downloaded here - (http://hgdownload.cse.ucsc.edu/goldenPath/hg19/encodeDCC/wgEncodeRegTfbsClustered/wgEncodeRegTfbsClusteredV3.bed.gz). Density plots describing the distributions of methylation at ChIP-seq peaks were plotted using the methDensityPlot function from aaRon (https://github.com/astatham/aaRon).

Heatmaps of the enrichment of histone ChIP-seq surrounding SA LMR/UMR/DMVs were created using Replitools (Statham et al. 2010).

Plotting methylation data

Aggregated DNA methylation profiles were plotted using Loess regression. Default settings except span = 0.2 was used.

RNA-seq analysis

Alignment/Mapping & Counting:

Adapters from the sequencing reads were trimmed using Trimmomatic (v0.33) (Bolger et al. 2014). Fastq sequences were then quality checked using FastQC bioinformatics tool (http://www.bioinformatics.babraham.ac.uk/projects/fastqc). Reads were then mapped to the human genome (hg19) using custom bioinformatics pipeline NGSANE (Buske et al. 2014), which utilizes TopHat (Trapnell et al. 2013) (TopHat v2.0.6) to perform the alignment in a strand specific manner. It is to be noted that TopHat was run using default parameters with options set as "fr-firststand" to specify library type and "-transcriptome-index" set to known transcriptome built from RefSeq.

Following the mapping process, htseqcount feature from the bioinformatics tool htseq (Anders et al. 2015) was employed to count the number of uniquely mapped reads of genomic features such as genes and transcripts guided by the GENCODE GTF (v19) (Harrow et al. 2012) annotation with the following parameter (HTSEQCOUNT_UNIQUE = 1 and GTF = "gencode.v19.annotation.gtf").

Differential Expression:

Differential expression analysis for subcutaneous adipocytes vs visceral adipocytes was performed using edgeR bioconductor package (Robinson et al. 2010; McCarthy et al. 2012). Previous studies have suggested that edgeR is better suited for evaluating differential expression between biological

conditions utilizing read count of genomic regions of interest, such as genes and transcripts(Robinson et al. 2010; McCarthy et al. 2012). The generalized linear model (GLM) algorithm of edgeR is able to separate out technical variability and extract feature-wise variation between condition even only using a few biological replicates (50,51). The topTag() function of edgeR was used to identify differentially expressed (DE) genes/transcripts with a Benjamin-Hochberg (BH) FDR correction method. Only genes/transcripts with FDR value <0.05 have been considered as DE genes/transcripts.

GO Analysis:

Typically Gene Ontology analysis (GO) is used to understand the underlying biological phenomenon/functions. For this study we have used goseq (Young et al. 2010) to perform gene functional enrichment analysis as it has been shown to discount for gene length bias that may have affected any differential expression call. Goseq typically identifies the over-represented GO terms that are statistically significant having a p-value <0.05. We then have further applied BH method to account for multiple testing corrections and only have considered the GO terms with adjusted FDR < 0.05 for further investigation and biological implication. In order to aid biological interpretation of terms only GO terms with between 15- 500 genes in the category were used for generation of bar plots.

For GO analysis on methylation data GO-seq was modified to account for CpG density of regions tested. Only DMRs within the promoters (2kb either side of TSS) or genebody (-2kb of TSS up until transcriptional end site) were used in GO analyses. To objectively summarise DMR GO terms tree plots were generated using REViGO (13). The top 100 GO terms were used as input for this process.

DATA AVAILABILITY

Data: GSE110821

Supplementary Tables

Table Number	Title
S1	Subject characteristics and genomic data
S2	SAvsVA Differentially expressed genes, PolyA RNA-seq
S3	SAvsVA Differentially expressed genes, Whole RNA-seq
S4	Gene Ontology of differentially expressed genes
S5	VATvsVA Differentially-expressed genes, PolyA RNA-seq
S6	Gene Ontology of VATvsVA Differentially-expressed genes
S7	SAvsVA Differentially Methylated Regions (DMRs), wgbs – Hypermethylated in VA
S8	SAvsVA Differentially Methylated Regions (DMRs), wgbs – Hypermethylated in SA
S9	SAvsVA Differentially Methylated Regions - 450K
S10	Gene Ontology of Low Differentially Methylated Regions (Low DMRs)
S11	Gene Ontology of High Differentially Methylated Regions (High DMRs)
S12	Transcription Factor genes with DMRs within 2kb of transcription start site
S13	Differentially-expressed genes with promoter DMRs (Δme>0.2)
S14	DE/DM genes in processes important to adipocyte functioning
S15	VA Unmethylated Regions (UMRs)
S16	SA Unmethylated Regions (UMRs)
S17	PBL Unmethylated Regions (UMRs)
S18	VA Low-Methylated Regions (LMRs)
S19	SA Low-Methylated Regions (LMRs)
S20	PBL Low-Methylated Regions (LMRs)
S21	DNA methylation valleys (DMVs)
S22	Gene Ontology of DMRs UMRs LMRs
S23	TF binding to DMRs, UMRs and LMRs
S24	Ratio SAVA_UMRs or LMRs relative to PBL
S25	Differential LMRs VA and SA
S26	TF Binding to Differential SA-LMRs
S27	TF Binding to Differential VA-LMRs
S28	Ratio of TF binding in High DMRs vs Low DMRs

Glossary for column headers in tables

Header		Tables
Chr, Start, End	Positions based on Hg19,	All
nCG segment	Number of CpGs in region covered by minimum number of reads	S15 – S20
nCG	Number of CpGs in region	S7-9, S15 – S20, S25
T	Total number of reads to the region	S15 – S20
M	Total number of reads to the region without conversion of the C	S15 – S20
pmeth	Mean methylation of the region	S15 – S20
Median meth	Median methylation of the region	S15 – S20
Type	UMR or LMR	S15 – S21
nGeneTSS	Number of TSS's overlapped by element	S7,S8, S15 – S21, S25
nProtGeneTSS	Number of protein coding gene TSS's overlapped by element	S12-S21, S25

distanceTSS, distanceTSS_Prot	Distance to nearest TSS, or protein coding gene TSS	S7,S8,S15 – S21,S25
TSS, TSS_prot	Index number for TSS	S7,S8, S15 – S21, S25
Tx-id, tx-id_prot	Gencode transcript ID	S15 – S21 S7,S8,,S13,S14,S17,S25
Gene_id, gene_id_prot	Gencode gene id, or protein-coding gene id	S7,S8,S15 – S21,S25
Gene_name, gene_name_prot	Name of nearest gene or protein-coding gene	S7,S8,S15 – S21,S25
distanceCpGi	Distance to nearest CpG island	S7,S8,S15 – S21,S25
CpGi	Index number of CpG island	S7,S8,S15 – S21,S25
Promoter, gene_body, intergenic (_prot)	Proportion of element within the promoter (+/- 2kb of TSS), gene-body or intergenic relative to nearest gene (or nearest protein-coding gene)	S7,S8,S15 – S21 S13,S14,S17,S25
CpGisland, CpG shores, non-CpG	Proportion of element overlapping a CpG island, CpG shore (+/- 2kb of island) or neither	S7,S8,S15 – S21,S25
Num_UMR_Genes_InCat	Number of genes, including 2 kb upstream of TSS, within GO category containing at least one UMR	S22
Num_LMR_Genes_InCat	Number of genes, including 2 kb upstream of TSS, within GO category containing at least one LMR	S22
Num_DMV_Genes_InCat	Number of genes, including 2 kb upstream of TSS, within GO category overlapping at least one DMV	S22
Num_DMR_Genes_InCat	Number of genes, including 2 kb upstream of TSS, within GO category containing at least one DMR	S10,S11
Category	GO category as defined at *www.geneontology.org/*	S11,S19,S22
Ontology	MF = Molecular Function; BP = Biological Process; CC = Cellular Compartment	S10,S11,S19,S22
maxbetafc	Maximum difference in beta value (SA minus VA) for a probe within region	S9
minfdr	minimum adjusted p value	S9
Stouffer	Stouffer combined p value for probes in region	S9
Rank	Rank of DMR ordered by absolute value of areaStat	S7,S8
invdensity		S7,S8,S25
areaStat	Sum of t-statistics for CpGs within region	S7,S8,S25
maxStat		S7,S8,S25
		S7,S8,S25
Tstat.sd		S7,S8,S25
Gene-ID	ENSEMBL gene ID	S2,S3,S5,S25
RPKM	Reads per kilobase (of gene length) per million mapped reads	S2,S3,S5
LogCPM		S2,S3,S5
LogFC	Log to base 2 of ratio of mean gene expression levels	S2,S3,S5

Result	Expression significantly (FDR<0.05) UP or DOWN with respect to VA. No Test – read counts below threshold. No change – sufficient read counts, but FDR > 0.05.	S2,S3,S5,S7,S8,S25

SUPPLEMENTARY REFERENCES

Able AA, Burrell JA, Stephens JM. 2017. STAT5-Interacting Proteins: A Synopsis of Proteins that Regulate STAT5 Activity. *Biology (Basel)* **6**.

Anders S, Pyl PT, Huber W. 2015. HTSeq--a Python framework to work with high-throughput sequencing data. *Bioinformatics* **31**: 166-169.

Arner P. 2005. Human fat cell lipolysis: biochemistry, regulation and clinical role. *Best Pract Res Clin Endocrinol Metab* **19**: 471-482.

Bailey SD, Zhang X, Desai K, Aid M, Corradin O, Cowper-Sal Lari R, Akhtar-Zaidi B, Scacheri PC, Haibe-Kains B, Lupien M. 2015. ZNF143 provides sequence specificity to secure chromatin interactions at gene promoters. *Nature communications* **2**: 6186.

Bolger AM, Lohse M, Usadel B. 2014. Trimmomatic: a flexible trimmer for Illumina sequence data. *Bioinformatics* **30**: 2114-2120.

Burger L, Gaidatzis D, Schubeler D, Stadler MB. 2013. Identification of active regulatory regions from DNA methylation data. *Nucleic acids research* **41**: e155.

Buske FA, French HJ, Smith MA, Clark SJ, Bauer DC. 2014. NGSANE: a lightweight production informatics framework for high-throughput data analysis. *Bioinformatics* **30**: 1471-1472.

Byun K, Gil SY, Namkoong C, Youn BS, Huang H, Shin MS, Kang GM, Kim HK, Lee B, Kim YB et al. 2014. Clusterin/ApoJ enhances central leptin signaling through Lrp2-mediated endocytosis. *EMBO Rep* **15**: 801-808.

Chen YA, Lemire M, Choufani S, Butcher DT, Grafodatskaya D, Zanke BW, Gallinger S, Hudson TJ, Weksberg R. 2013. Discovery of cross-reactive probes and polymorphic CpGs in the Illumina Infinium HumanMethylation450 microarray. *Epigenetics : official journal of the DNA Methylation Society* **8**: 203-209.

Chikina MD, Troyanskaya OG. 2012. An effective statistical evaluation of ChIPseq dataset similarity. *Bioinformatics* **28**: 607-613.

Clark SJ, Statham A, Stirzaker C, Molloy PL, Frommer M. 2006. DNA methylation: bisulphite modification and analysis. *Nature protocols* **1**: 2353-2364.

Cole BK, Morris MA, Grzesik WJ, Leone KA, Nadler JL. 2012. Adipose tissue-specific deletion of 12/15-lipoxygenase protects mice from the consequences of a high-fat diet. *Mediators Inflamm* **2012**: 851798.

Dalgaard K, Landgraf K, Heyne S, Lempradl A, Longinotto J, Gossens K, Ruf M, Orthofer M, Strogantsev R, Selvaraj M et al. 2016. Trim28 Haploinsufficiency Triggers Bi-stable Epigenetic Obesity. *Cell* **164**: 353-364.

Dick KJ, Nelson CP, Tsaprouni L, Sandling JK, Aïssi D, Wahl S, Meduri E, Morange P-E, Gagnon F, Grallert H et al. 2014. DNA methylation and body-mass index: a genome-wide analysis. *The Lancet* **383**: 1990-1998.

Dihazi GH, Jahn O, Tampe B, Zeisberg M, Muller C, Muller GA, Dihazi H. 2015. Proteomic analysis of embryonic kidney development: Heterochromatin proteins as epigenetic regulators of nephrogenesis. *Sci Rep* **5**: 13951.

Eguchi J, Wang X, Yu S, Kershaw EE, Chiu PC, Dushay J, Estall JL, Klein U, Maratos-Flier E, Rosen ED. 2011. Transcriptional control of adipose lipid handling by IRF4. *Cell metabolism* **13**: 249-259.

Ellis BC, Graham LD, Molloy PL. 2014. CRNDE, a long non-coding RNA responsive to insulin/IGF signaling, regulates genes involved in central metabolism. *Biochimica et biophysica acta* **1843**: 372-386.

Friedman JR, Kaestner KH. 2006. The Foxa family of transcription factors in development and metabolism. *Cell Mol Life Sci* **63**: 2317-2328.

Gao H, Mejhert N, Fretz JA, Arner E, Lorente-Cebrian S, Ehrlund A, Dahlman-Wright K, Gong X, Stromblad S, Douagi I et al. 2014. Early B cell factor 1 regulates adipocyte morphology and lipolysis in white adipose tissue. *Cell metabolism* **19**: 981-992.

Gavin KM, Cooper EE, Hickner RC. 2013. Estrogen receptor protein content is different in abdominal than gluteal subcutaneous adipose tissue of overweight-to-obese premenopausal women. *Metabolism* **62**: 1180-1188.

Gealekman O, Guseva N, Hartigan C, Apotheker S, Gorgoglione M, Gurav K, Tran KV, Straubhaar J, Nicoloro S, Czech MP et al. 2011. Depot-specific differences and insufficient subcutaneous adipose tissue angiogenesis in human obesity. *Circulation* **123**: 186-194.

Gehrke S, Brueckner B, Schepky A, Klein J, Iwen A, Bosch TC, Wenck H, Winnefeld M, Hagemann S. 2013. Epigenetic regulation of depot-specific gene expression in adipose tissue. *PloS one* **8**: e82516.

Griffin MJ, Zhou Y, Kang S, Zhang X, Mikkelsen TS, Rosen ED. 2013. Early B-cell factor-1 (EBF1) is a key regulator of metabolic and inflammatory signaling pathways in mature adipocytes. *The Journal of biological chemistry* **288**: 35925-35939.

Hansen KD, Langmead B, Irizarry RA. 2012. BSmooth: from whole genome bisulfite sequencing reads to differentially methylated regions. *Genome biology* **13**: R83.

Harrow J, Frankish A, Gonzalez JM, Tapanari E, Diekhans M, Kokocinski F, Aken BL, Barrell D, Zadissa A, Searle S et al. 2012. GENCODE: the reference human genome annotation for The ENCODE Project. *Genome research* **22**: 1760-1774.

Hogan MC, Griffin MD, Rossetti S, Torres VE, Ward CJ, Harris PC. 2003. PKHDL1, a homolog of the autosomal recessive polycystic kidney disease gene, encodes a receptor with inducible T lymphocyte expression. *Human molecular genetics* **12**: 685-698.

Hovestadt V, Jones DT, Picelli S, Wang W, Kool M, Northcott PA, Sultan M, Stachurski K, Ryzhova M, Warnatz HJ et al. 2014. Decoding the regulatory landscape of medulloblastoma using DNA methylation sequencing. *Nature* **510**: 537-541.

Kakui Y, Uhlmann F. 2017. SMC complexes orchestrate the mitotic chromatin interaction landscape. *Curr Genet* doi:10.1007/s00294-017-0755-y.

Krueger F, Andrews SR. 2011. Bismark: a flexible aligner and methylation caller for Bisulfite-Seq applications. *Bioinformatics* **27**: 1571-1572.

Lawrence M, Huber W, Pages H, Aboyoun P, Carlson M, Gentleman R, Morgan MT, Carey VJ. 2013. Software for computing and annotating genomic ranges. *PLoS Comput Biol* **9**: e1003118.

Locke AE Kahali B Berndt SI Justice AE Pers TH Day FR Powell C Vedantam S Buchkovich ML Yang J et al. 2015. Genetic studies of body mass index yield new insights for obesity biology. *Nature* **518**: 197-206.

Ma X, Yang P, Kaplan WH, Lee BH, Wu LE, Yang JY, Yasunaga M, Sato K, Chisholm DJ, James DE. 2014. ISL1 regulates peroxisome proliferator-activated receptor gamma activation and early adipogenesis via bone morphogenetic protein 4-dependent and -independent mechanisms. *Molecular and cellular biology* **34**: 3607-3617.

Macartney-Coxson D, Benton MC, Blick R, Stubbs RS, Hagan RD, Langston MA. 2017. Genome-wide DNA methylation analysis reveals loci that distinguish different types of adipose tissue in obese individuals. *Clin Epigenetics* **9**.

Maekawa T, Jin W, Ishii S. 2010. The role of ATF-2 family transcription factors in adipocyte differentiation: antiobesity effects of p38 inhibitors. *Molecular and cellular biology* **30**: 613-625.

Marquez MP, Alencastro F, Madrigal A, Jimenez JL, Blanco G, Gureghian A, Keagy L, Lee C, Liu R, Tan L, Deignan K, Armstrong B, Zhao Y. 2017. The Role of Cellular Proliferation in Adipogenic Differentiation of Human Adipose Tissue-Derived Mesenchymal Stem Cells. Stem Cells Dev. 26:1578-1595.

McCarthy DJ, Chen Y, Smyth GK. 2012. Differential expression analysis of multifactor RNA-Seq experiments with respect to biological variation. *Nucleic acids research* **40**: 4288-4297.

Peters TJ, Buckley MJ, Statham AL, Pidsley R, Samaras K, Lord RV, Clark SJ, Molloy PL. 2015. De novo identification of differentially methylated regions in the human genome. *Epigenet Chromatin* **8**.

Pidsley R, CC YW, Volta M, Lunnon K, Mill J, Schalkwyk LC. 2013. A data-driven approach to preprocessing Illumina 450K methylation array data. *BMC genomics* **14**: 293.

Reynisdottir S, Wahrenberg H, Carlstrom K, Rossner S, Arner P. 1994. Catecholamine resistance in fat cells of women with upper-body obesity due to decreased expression of beta 2-adrenoceptors. *Diabetologia* **37**: 428-435.

Ritchie ME, Phipson B, Wu D, Hu Y, Law CW, Shi W, Smyth GK. 2015. limma powers differential expression analyses for RNA-sequencing and microarray studies. *Nucleic acids research* **43**: e47.

Roadmap Epigenomics C, Kundaje A, Meuleman W, Ernst J, Bilenky M, Yen A, Heravi-Moussavi A, Kheradpour P, Zhang Z, Wang J et al. 2015. Integrative analysis of 111 reference human epigenomes. *Nature* **518**: 317-330.

Robinson MD, McCarthy DJ, Smyth GK. 2010. edgeR: a Bioconductor package for differential expression analysis of digital gene expression data. *Bioinformatics* **26**: 139-140.

Rodbell M. 1964. Metabolism of Isolated Fat Cells. I. Effects of Hormones on Glucose Metabolism and Lipolysis. *The Journal of biological chemistry* **239**: 375-380.

Schmidt SF, Jorgensen M, Chen Y, Nielsen R, Sandelin A, Mandrup S. 2011. Cross species comparison of C/EBPalpha and PPARgamma profiles in mouse and human adipocytes reveals interdependent retention of binding sites. *BMC genomics* **12**: 152.

Shungin D Winkler TW Croteau-Chonka DC Ferreira T Locke AE Magi R Strawbridge RJ Pers TH Fischer K Justice AE et al. 2015. New genetic loci link adipose and insulin biology to body fat distribution. *Nature* **518**: 187-196.

Slieker RC, Bos SD, Goeman JJ, Bovee JV, Talens RP, van der Breggen R, Suchiman HE, Lameijer EW, Putter H, van den Akker EB et al. 2013. Identification and systematic annotation of tissue-specific differentially methylated regions using the Illumina 450k array. *Epigenetics Chromatin* **6**: 26.

Statham AL, Strbenac D, Coolen MW, Stirzaker C, Clark SJ, Robinson MD. 2010. Repitools: an R package for the analysis of enrichment-based epigenomic data. *Bioinformatics* **26**: 1662-1663.

Supek F, Bosnjak M, Skunca N, Smuc T. 2011. REVIGO summarizes and visualizes long lists of gene ontology terms. *PloS one* **6**: e21800.

Thorvaldsdottir H, Robinson JT, Mesirov JP. 2013. Integrative Genomics Viewer (IGV): high-performance genomics data visualization and exploration. *Brief Bioinform* **14**: 178-192.

Trapnell C, Hendrickson DG, Sauvageau M, Goff L, Rinn JL, Pachter L. 2013. Differential analysis of gene regulation at transcript resolution with RNA-seq. *Nature biotechnology* **31**: 46-53.

Weber M, Hellmann I, Stadler MB, Ramos L, Paabo S, Rebhan M, Schubeler D. 2007. Distribution, silencing potential and evolutionary impact of promoter DNA methylation in the human genome. *Nature genetics* **39**: 457-466.

Wolfs MG, Rensen SS, Bruin-Van Dijk EJ, Verdam FJ, Greve JW, Sanjabi B, Bruinenberg M, Wijmenga C, van Haeften TW, Buurman WA et al. 2010. Co-expressed immune and metabolic genes in visceral and subcutaneous adipose tissue from severely obese individuals are associated with plasma HDL and glucose levels: a microarray study. *BMC medical genomics* **3**: 34.

Xi D, Gandhi N, Lai M, Kublaoui BM. 2012. Ablation of Sim1 neurons causes obesity through hyperphagia and reduced energy expenditure. *PloS one* **7**: e36453.

Xu Z, Yu S, Hsu CH, Eguchi J, Rosen ED. 2008. The orphan nuclear receptor chicken ovalbumin upstream promoter-transcription factor II is a critical regulator of adipogenesis. *Proceedings of the National Academy of Sciences of the United States of America* **105**: 2421-2426.

Young MD, Wakefield MJ, Smyth GK, Oshlack A. 2010. Gene ontology analysis for RNA-seq: accounting for selection bias. *Genome biology* **11**: R14.

Zhang H, Chen X, Sairam MR. 2012. Novel genes of visceral adiposity: identification of mouse and human mesenteric estrogen-dependent adipose (MEDA)-4 gene and its adipogenic function. *Endocrinology* **153**: 2665-2676.

Zhou J, Wang S, Qi Q, Yang X, Zhu E, Yuan H, Li X, Liu Y, Li X, Wang B. 2017. Nuclear factor I-C reciprocally regulates adipocyte and osteoblast differentiation via control of canonical Wnt signaling. *FASEB journal : official publication of the Federation of American Societies for Experimental Biology* **31**: 1939-1952.

Ziller MJ, Hansen KD, Meissner A, Aryee MJ. 2015. Coverage recommendations for methylation analysis by whole-genome bisulfite sequencing. *Nature methods* **12**: 230-232, 231 p following 232.
